# 3D-Printed MOF Monoliths: Fabrication Strategies and Environmental Applications

**DOI:** 10.1007/s40820-024-01487-1

**Published:** 2024-08-15

**Authors:** Hossein Molavi, Kamyar Mirzaei, Mahdi Barjasteh, Seyed Yahya Rahnamaee, Somayeh Saeedi, Aliakbar Hassanpouryouzband, Mashallah Rezakazemi

**Affiliations:** 1 Department of Chemistry, Institute for Advanced Studies in Basic Science (IASBS), Zanjan, 45137-66731 Iran; 2 Department of Chemical and Biological Engineering, University at Buffalo, The State University of New York, Buffalo, NY 14260 USA; 3 Center for Nano-Science and Nanotechnology, Institute for Convergence Science & Technology, Sharif University of Technology, Tehran, 15614 Iran; 4 Department of Biomedical Engineering, Amirkabir University of Technology (Tehran Polytechnic), Hafez Ave., P.O.Box 15875-4413, Tehran, Iran; 5 Grant Institute, School of Geosciences, University of Edinburgh, West Main Road, Edinburgh, EH9 3FE UK; 6 Faculty of Chemical and Materials Engineering, Shahrood University of Technology, Shahrood, P.O. Box 3619995161, Iran

**Keywords:** MOFs, 3D-printing, Environmental remediation, Shaping, Monoliths

## Abstract

Metal–organic frameworks (MOFs) have been extensively considered as one of the most promising types of porous and crystalline organic–inorganic materials, thanks to their large specific surface area, high porosity, tailorable structures and compositions, diverse functionalities, and well-controlled pore/size distribution. However, most developed MOFs are in powder forms, which still have some technical challenges, including abrasion, dustiness, low packing densities, clogging, mass/heat transfer limitation, environmental pollution, and mechanical instability during the packing process, that restrict their applicability in industrial applications. Therefore, in recent years, attention has focused on techniques to convert MOF powders into macroscopic materials like beads, membranes, monoliths, gel/sponges, and nanofibers to overcome these challenges.Three-dimensional (3D) printing technology has achieved much interest because it can produce many high-resolution macroscopic frameworks with complex shapes and geometries from digital models. Therefore, this review summarizes the combination of different 3D printing strategies with MOFs and MOF-based materials for fabricating 3D-printed MOF monoliths and their environmental applications, emphasizing water treatment and gas adsorption/separation applications. Herein, the various strategies for the fabrication of 3D-printed MOF monoliths, such as direct ink writing, seed-assisted in-situ growth, coordination replication from solid precursors, matrix incorporation, selective laser sintering, and digital light processing, are described with the relevant examples. Finally, future directions and challenges of 3D-printed MOF monoliths are also presented to better plan future trajectories in the shaping of MOF materials with improved control over the structure, composition, and textural properties of 3D-printed MOF monoliths.

## Highlights

- Challenges and future directions for 3D-printed metal-organic frameworks (MOFs) monoliths in environmental applications are discussed.
- Various strategies for fabrication of 3D-printed MOF monoliths are summarized.
- Advancements in 3D printing enable customizable and high-performance MOF monoliths.
- 3D orienting of MOFs opens avenues for applications in water treatment and gas adsorption.

## Graphical abstract

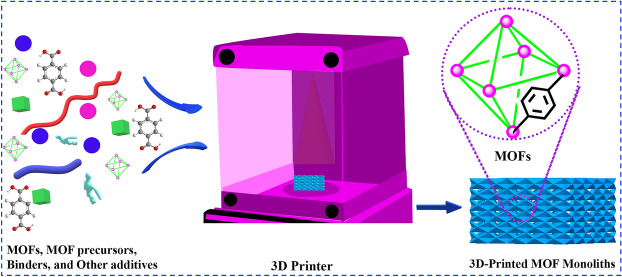

## Introduction

Metal–organic frameworks (MOFs) (also known as porous coordination polymers (PCPs)) are an attractive family of organic–inorganic hybrid and crystalline porous materials that are constructed through coordination bonds of metal ions/clusters and organic ligands [1–3]. This emerging class of porous materials has garnered much attention since 1995, when Yaghi initially described MOFs [4] and further developed them in the 2000s [5]. In general, the MOFs framework, functionality, and pore structure and environment could be accurately tuned by elaborately selecting favorable metal salt and organic linker precursors, post-synthetic modification of MOF skeletons, and logical design of topological structures [6]. Therefore, they show outstanding physiochemical characteristics like abundant active sites, remarkable specific surface area, low density, permanent porosity, and different topological structures [7, 8].

Owing to their fascinating properties, MOFs have found several applications in different fields, including adsorption/separation, sensors, energy storage, nonlinear optics, drug delivery, chromatography, supercapacitors, catalysis, etc. [9–15]. In particular, MOFs encourage the use of porous materials for water purification owing to their exceptional properties and precisely defined apertures that can be well controlled over a wide range to allow shape and size selectivity toward different water pollutants [16–19]. Moreover, the complex pores in the structure of MOFs provide active sites for isolation, coupling, and cooperation functionalities, which lead to the high catalytic activity of MOFs [20]. The emergence of a highly water-stable MOF family, Zr-based MOF family, specifically UiO-66 (University of Oslo), has opened up a new window in the design and practical water treatment applications of MOFs [21].

Until now, most of the reported MOFs have been in powder form and usually prepared in a small scale [22]. Most current MOF manufacturing methods are costly, time-consuming, environmentally hazardous, and energy-intensive due to needing a large volume of hazardous and high-boiling point organic solvents and producing waste by-products [23]. Thus, developing new synthetic methods is essential to transfer small-scale production to large-scale [24]. For instance, Crawford et al. [25] reported that extrusion methods are able to produce MOFs on large-scale under solvent-free or low-solvent conditions. This strategy was also applied to synthesis many MOFs via a green, continuous, and large-scale method [26].

On the other hand, the applicability of MOF powders in industrial and large-scale applications is limited by different challenges, including abrasion, dustiness, low packing densities, clogging, mass/heat transfer limitation, environmental pollution, and mechanical instability during the packing process [27, 28]. Moreover, the major challenge of these materials is their low chemical, thermal, and mechanical stability compared to other typical porous materials [29]. For example, it was reported that the mass transfer resistance increased with increasing column time when using packed HKUST-1 powder for CH_4_ capture due to the significant decrease in pressure [30]. Moreover, the MOF powder as a catalyst or adsorbent showed various disadvantages like dust formation, secondary pollution, difficulty separating from the reaction environment, and difficulty in regeneration and recycling [31, 32].

### Shaping of MOF Powders

MOF powders must be incorporated into or immobilized onto packed objects with predetermined dimensions and sizes to address the aforementioned obstacles [33, 34]. Individual crystallites are compacted into millimeter-sized objects during this process, referred to as shaping [35]. Compared to pure MOF powders, the shaping or densification of MOF powders into an object shows different advantages, such as improved mechanical stability, enhanced structural stability, and increased packing density [36]. More importantly, it was reported that shaping MOF powders into millimeter-sized objects suitable for industrial applications is an essential process towards the commercialization of this kind of porous material [37, 38]. Previous studies have reported several techniques for shaping MOF powders into granules, beads, pellets, monoliths, etc. [39–41]. Every shaping method imparts distinct properties to the final structures regarding size, morphology, and manifestation for a particular purpose [42].

To date, different approaches, such as in situ growth, direct mixing, palletization, and deposition of MOF powders with various polymer matrices, foams, cotton, or other porous substrates, have been applied to convert MOF powders into macroscopic materials like beads, membranes, gel/sponges, and nanofibers [43, 44]. For instance, granulation or palletization is frequently used to convert MOF powders to macrostructures, in which the need for high-pressure for pressing is a critical challenge of this strategy. The pressing pressure of this strategy may reduce the porosity of MOFs and their active sites, thereby reducing their performances in different applications. Solvents or binders (e.g., organic binders, inorganic binders, etc.) can be used during the palletization process to overcome these challenges and reduce the pressing pressure. It was found that binders remarkably improve the mechanical stability of resulting pellet, while they can block the pores of MOFs and decrease their surface areas. Accordingly, different review papers have been reported that summarized the shaping methods of MOFs in view of fundamental and technical aspects and their limitations [38, 45].

### 3D Printing Technology

Three-dimensional (3D) printing technology, as an additive manufacturing bottom-up technology, has received much attention as an attractive and innovative technology since it can create numerous high-resolution structures using digital models [46–48]. This strategy can shape MOF powders into adjustable 3D MOF structures, which significantly increases their applicability in industrial applications [49, 50]. Compared to the conventional shaping techniques, 3D printing has the following advantages: (i) the specific monoliths with complex shape and structures could be simply designed through many computer-aided modeling softwares, (ii) the adaption of the 3D-printed monoliths can be simply achieved through the printer parameters and design model, making it an attractive and versatile process, (iii) extending the applications of MOFs in large-scale practical applications, (iv) unlike other shaping methods, the fabricated 3D-printed MOF monoliths via this strategy usually have interconnected channels, which facilitates the transfer of heat and mass during the operation conditions, (v) in comparison to the granulation process, 3D printing technology can produce regulated channels within the fabricated monoliths, which decreases the pressure drop as well as lower energy consumption, (vi) more uniformly combined different materials, resulting in the presence of more active sites on the surface of 3D-printed monoliths, and (vii) preserving the MOFs' active sites for better interactions with different materials in specific applications like environmental remediation [51–53].

So far, 3D printing has been applied to convert MOF-based materials into tailorable shapes for various applications, including water treatment [54], gas separation [55], drug delivery [56], pollutant detection [57, 58], gas storage [59], moisture sensing [60], catalysis [61], batteries [62], and biomedicine [63]. Excellent review papers summarize the recent advances of 3D printing technology in different applications like separation processes and catalytic applications [52, 53, 64]. For example, Zhu et al. [52] summarized the details of different 3D printing strategies, including vat photopolymerization-based, extrusion-based, and powder-based strategies that can be applied in catalytic applications. Subsequently, they classified different 3D printing technologies based on the printing of various adsorption-based materials. Then, the effect of different influencing parameters on the structure of resulting 3D-printed monoliths as well as the influence of 3D-printing structures on the adsorption process, were comprehensively discussed [53]. Moreover, Liu et al. [64] comprehensively discussed the influencing parameters on the structure of resulting 3D-printed metal/covalent organic frameworks (M/COFs) monoliths. Then introduced the recent progress for preserving the favorable microstructure properties of M/COFs in resulting 3D-printed M/COFs that are very important in their applications.

However, a comprehensive review of the characteristics and challenges of 3D printing technology for shaping MOFs has not been reported. Therefore, the current review presents general reports on recent developments in designing and fabricating various kinds of 3D-printed MOF monoliths. This review discusses the specific details of the whole fabrication strategy for converting MOF powders into 3D-printed MOF monoliths, including the impact of ink rheology on the textural properties of the final object and the appropriate choice of a MOF, binder, plasticizer, and solvent for printing. Then, each 3D printing technology's advantages and disadvantages are summarized per their requirements. Subsequently, we reviewed the environmental remediation applications of the 3D-printed MOF monoliths regarding gas separation and water purification. Ultimately, the challenges and future prospects of this area are described in order to suggest a direction for future research. We hope that this review will provide deep and novel insights into the relationship between the performance and microstructure of 3D-printed MOF monoliths, thus hastening the evolution of this hierarchical porous material in large-scale industrial applications.

## Strategies for Converting MOF Powders into the 3D-Printed MOF Monoliths

Based on the technical processes, 3D printing technology can be classified into different categories, including (i) extrusion of materials (e.g., direct ink writing (DIW) and fused deposition modeling (FDM)), (ii) vat polymerization (e.g., selective laser sintering (SLS) and digital light processing (DLP)), (iii) powder bed fusion (e.g., SLS, electron beam melting, and selective laser melting (SLM)), (iv) laminated object manufacturing, (v) material jetting (e.g., aerosol jet printing, inkjet printing, and electrical field driven jetting), (vi) directed energy deposition, and (vii) binder jetting [55, 65–68]. For instance, da Luz et al. [67] reported printing photoluminescent Lanthanide-Organic Frameworks (Ln-MOFs) over the foils of paper and plastic via a typical inkjet printer. Therein, Ln-MOF ink was applied to create color images that could only be seen when exposed to ultraviolet (UV) light. The authors reported that this fabrication strategy can open up a new window to explore the applications of Ln-MOF materials in technological uses like optical devices (e.g., lab-on-a-chip) and optical document authentication. Inspired by this work, different research groups prepared 3D-printed MOF monoliths via this strategy and applied them for various applications, including electrocatalysts [69], sensors [70], bio-related applications [71], etc.

**Fig. 1:**
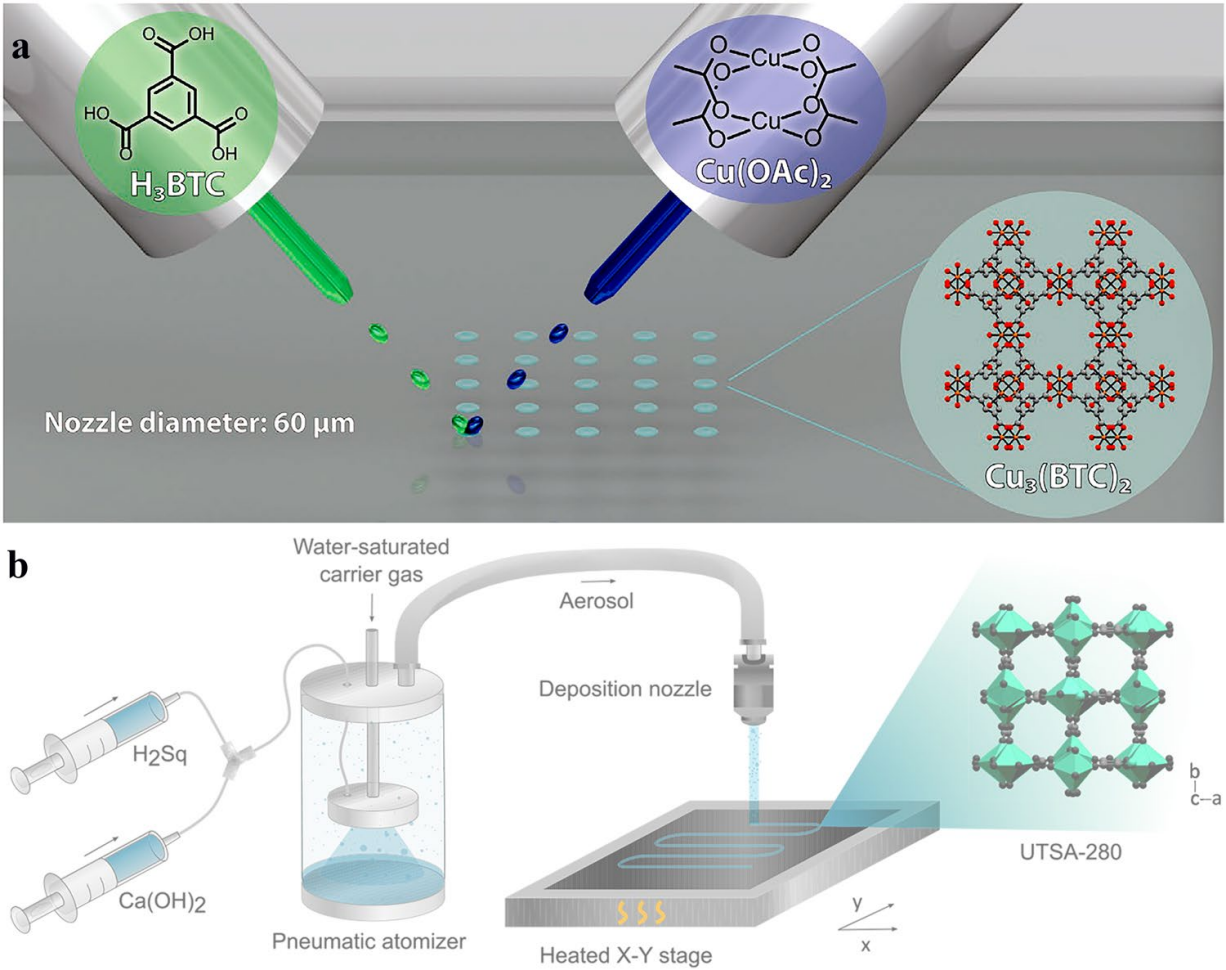
Schematic illustrating **a** preparation of Cu_3_(BTC)_2_ MOF onto a substrate via reactive inkjet printing strategy. Reprinted with permission from Ref. [72]. Copyright 2023, John Wiley & Sons. **b** Aerosol jet printing setup applied to deposit UTSA-280 onto a substrate. Reprinted with permission from Ref. [74]. Copyright 2022, American Chemical Society

Reactive inkjet printing is a new manufacturing method for decorating the substrate surface with MOFs, in which the desired MOFs were prepared in situ by jetting the precursor solutions of MOFs onto a substrate (Fig. 1a). This manufacturing strategy dissolves different challenges of preparing inks/suspensions, including stability, nozzle-clogging, and life-time [72, 73]. For the generation of droplets in this strategy, the surface tension of near-liquid ink and its viscosity must be precisely controlled. Due to the dissipation of viscous kinetic energy at the nozzle, the droplet cannot be ejected when the ink viscosity is too high. Conversely, the surface tension of droplets is a key parameter that can control the formation of separate droplets from the ink. Accordingly, the final diameter of the droplet deposited onto the substrate's surface determines the resolution limit of this 3D printing strategy [69].

Aerosol jet printing is another emerging contactless direct-write method that was applied to directly deposit a thin layer of MOFs onto the surface of substrates from the aerosol of aqueous MOF precursor solutions (Fig. 1b) [74]. Although this strategy has most of the advantages of the inkjet printing strategy, the viscosity of ink does not play the key role, so no ink optimization is required. Unlike other printing strategies, this strategy can be applied to form a specific pattern on any substrate directly. The resolution of printed patterns via this strategy significantly depends on the jet shape and applying efficient aerodynamic focusing can reach down to 50 µm [75].

The DIW strategy is usually applied for printing different materials like ceramics, gel, polymers, carbon-based materials, plastics, etc. [76]. This strategy demands that the printing ink has suitable fluidity in the extrusion process to guarantee smooth extrusion and adequate viscosity after printing to retain the shape. Accordingly, a viscoelastic ink with appropriate rheological properties such as yield stress, viscosity, elastic/storage (G′), and viscous/loss (G″) moduli must first be produced. For instance, ink with extremely high viscosity can block the nozzle, thereby needing higher printing pressures. The resolution of printed structures via this strategy is controlled by a combination of applied pressure, the properties of extrusion ink, and the nozzle diameter [77]. Thus, an ink with optimized elastic behavior and viscosity is desirable for DIW strategy, particularly showing shear-thinning behavior, in which the viscosity reduces as the shear rate increases [50].

An ideal ink is usually produced by mixing the active materials (herein MOFs) with a solvent, binder, and plasticizer, in which the added binder bonds the active materials during the printing process, while the plasticizer is used to regulate the rheological properties of ink to facilitate the printing process [78]. This strategy has the advantages of low cost, simple operation, and operating at room temperature, while its main disadvantage is the relatively poor accuracy. Although a self-standing 3D structure is usually fabricated via this strategy, post-processing steps (e.g., drying, heating, and sintering) are generally required to improve its mechanical and functional properties.

The FDM strategy was invented in 1989 [79] and gradually developed; it is currently one of the most widespread and popular 3D printing technologies. It operates by heating the thermoplastic filaments to a semi-molten state, in which the filaments enter the heating chamber, melt at a relatively high temperature, and then extrude from the nozzle tip. Finally, the extruded materials are quickly cured to produce a uniform solid layer. The desired 3D structure was constructed through a layer-by-layer deposition mechanism by repeating these processes [80].

Compared to DIW, this strategy restricts using only a few printable polymer-based materials with relatively low melting temperature and suitable thixotropic characteristics to meet the requirements for FDM strategy. To date, different thermoplastic polymers such as poly (lactic acid) (PLA), acrylonitrile butadiene styrene (ABS), thermoplastic polyurethane (TPU), polyamide (PA), and polycarbonate were printed via this strategy [81]. Moreover, applicable materials for this strategy must be a solid filament with a specific length and width. Like DIW, the fabricated 3D structures via FDM strategy need post-printing treatment processes to improve their mechanical and functional properties. Nevertheless, this 3D printing strategy is independent of the rheological characteristics of filament/ink, however, it depends on the fabrication of the thermoplastic composite filaments instead [82]. Although the printing accuracy was better than DIW strategy, its operating temperature was higher, and the material printability was narrow [83].

In contrast to the example of MOF coating grown onto the external surface of 3D-printed monoliths using DIW strategy [84], the FDM strategy has been applied to produce 3D-printed monoliths with the complete incorporation of MOF nanoparticles inside a melting matrix of PLA, ABS [54, 85], or TPU [86]. Nevertheless, the porosity loss in FDM processable thermoplastic/MOF composites usually calls into doubt their further applicability for up-scaling. Unlike the FDM strategy, the DIW strategy has produced robust 3D-printed monoliths with easily accessible interior porosity that are suitable for various applications such as catalysis and adsorption. Additionally, up to now, several representative examples of 3D-printed MOF monoliths with high MOF contents appropriate for catalysis [61, 87], gas separation and storage [88, 89], energy storage [90], sensors [57, 58, 91], biomedicine [56, 63, 92], toxic materials degradation [93], and water treatment [94] have been reported.

According to the current literature, several well-known techniques for manufacturing of 3D-printed MOF monoliths have been developed in recent years, whose details have been comprehensively summarized in different review papers [50, 52, 53, 64]. However, this section discusses some of these strategies including (i) DIW, (ii) seed-assisted in situ growth, (iii) coordination replication from solid precursors, (iv) matrix incorporation, (v) SLS, and (vi) DLP. These techniques are usually classified by their material loading procedures, paste fabrication methods, and the physiochemical properties of the final monoliths; their advantages and disadvantages are summarized in Table 1. As a result, this section is focused on these fabrication strategies and summarizes some of the highlighted studies in this field.

**Table 1:** Comparison of the advantages and disadvantages of different 3D printing strategies

3D printing strategies	Advantages	Disadvantages	Most reported 3D-printed MOF monoliths
Direct ink writing	Simple Operating at room temperature Low cost Wide range of printable materials Fast production rate	-Clogging of printing nozzle -Possibility to partially disintegrate MOFs during the binding and densification processes -Pore blockage of MOFs -Needing a laborious procedure to form an ink suitable for printing -Low mechanical properties -Particle agglomeration in high MOF loadings -Need for rheological modifiers -Relatively poor accuracy -Needing post-processing steps	-UTSA-16(Co) -MOF-74(Ni) -UiO-66 -HKUST-1 -Co-MOFs -ZIF-8 -ZIF-67
Seed-assisted in situ growth	Producing 3D structure with good functionality Good interaction between MOFs and matrices Uniform distribution of MOFs within the matrices Good control over the nucleation and growth of MOFs	-Not suitable for high MOF loadings -Production of MOF phase with relatively poor crystallinity -Relatively time consuming -More waste production -Not very suitable for scale-up	-Cu-BTC MOFs -MOF-74 -Ln-MOFs -UTSA-16(Co) -HKUST-1 -ZIF-8 -Ce-MOFs
Coordination replication from solid precursors	Good control over the nucleation and growth of MOFs Good interaction between MOFs and matrices Uniform coatings of MOF particles Formation of homogeneous and continuous layers of MOFs with controlled thickness Exposure of more MOFs’ active sites on the surface of monoliths	-More waste production -Relatively time consuming -Low stability of some printable polymers under MOF synthesis condition -Not suitable for high MOF loadings -Low production rate -Production of MOF phase with relatively poor crystallinity	-ZIF-8 -Ca-MOFs
Matrix incorporation	Good interaction between MOFs and matrices Uniform distribution of MOFs within the matrices Good mechanical properties Low rheological limitations Low cost Fabricating MOF monoliths via a simple solvent-casting process	-Not suitable for high MOF loadings -Possibility of partial decomposition of the MOF crystals -Low production rate -Pore blockage of MOFs	-MOF-5 -MOF-74 -HKUST-1 -UiO-66
Selective laser sintering	High resolution Good control over the construction of MOF monoliths Good control over the physical characteristics of the fabricated monoliths Formation of micro-voids between MOFs and polymer powders High surface area Improving the exposure of MOF nanoparticles to the external ambient	-Narrow range of applicable materials -High cost -Low production rate -Operating at relatively high temperature -Low stability of some MOFs under laser sintering condition -Poor interfacial interactions between MOF and matrices	-HKUST-1 -NH_2_-MIL-101(Al) -MOF-801 -ZIF-67 -ZIF-8
Digital light processing	High resolution Good control over the construction of MOF monoliths Fast production rate Good control over the layer's thickness Suitable for rapid fabrication of MOF-MMMs	-Limited to photosensitive resin -High cost -Needing photoinitiators -Needing a light source -Low mechanical properties	-MIL-53(Al)-NH_2_ -HKUST-1

### Direct Ink Writing

The direct ink writing (DIW) strategy was the first and most important technique for producing 3D-printed monoliths. The 3D-printed monoliths are typically generated by synthesizing the MOF particles or using commercial MOF powders and suspending them in a paste containing a binder, co-binder, solvents, and plasticizer. Following the fabrication of suitable ink, the ink is normally rolled at room temperature over 1–2 days in the binding process to guarantee appropriate interfacial interaction among the different ingredients and yield great homogeneity [95]. Subsequently, in the densification step, the prepared ink is gradually heated to 313–333 K for several hours to completely remove the solvent and form a printable paste rheology [55, 56, 61, 63, 94]. However, it is important to note that this strategy is not always the best choice for fabricating 3D-printed MOF monoliths because some reported MOFs partially disintegrate during the binding and densification processes [96].

DIW strategy has attracted great consideration as it can form many 3D-printed structures from digital models [94, 97, 98]. However, one of the most important challenge of this strategy is accurate controlling the rheology of prepared ink to be printable and maintain its structure and shape after printing [99]. Moreover, the prepared filaments after extrusion also need adequate mechanical properties (e.g., storage modulus, yield stress, etc.) to allow for overhanging structure [100]. To date, different materials have been used as rheology modifiers to prepare suitable 3D-printed MOF monoliths, some of which were listed in Table 2. For instance, Rezaei and coworkers [55] have used bentonite clay as rheology modifier to prepare ink with appropriate viscoelastic properties for DIW of UTSA-16(Co) and MOF-74(Ni) into cylinders with a size of 15 mm, in which the fabricated 3D-printed MOF monoliths showed superior gas adsorption performance and structural stability than their powder counterparts. Similarly, Bouzga and coworkers [84] reported the preparation of 3D-printed UTSA-16 monoliths via a novel non-aqueous ink formulization using boehmite and hydroxypropyl cellulose as the rheology modifiers. It was reported that the existence of a binder matrix (boehmite and hydroxypropyl cellulose) not only regulated the rheology of the ink as well as its printability but also improved the dispersion of UTSA-16 nanoparticles in the fabricated 3D-printed monoliths. Based on the obtained results, the authors proposed that the fabricated 3D-printed UTSA-16 structure is an efficient CO_2_ capture adsorbent, as it showed high CO_2_ uptake and relatively good structural stability (negligible degradation) in the presence of humidity or water, mainly due to the presence of hydrophobic hydroxypropyl cellulose plasticizer, which repelled water molecules from the MOF center [101].

**Table 2:** Summary of chemical composition of different reported 3D-printed MOFs monoliths

Monoliths	Fabrication strategy	Binders	Additives	Applications	References
UTSA-16	DIW	Boehmite	Isopropyl alcohol (as a solvent) hydroxypropyl cellulose^a^	CO_2_ adsorption	[84]
ZIF-67	Stereolithography	PVDF	Acetone/DMF (as solvents)	Dye degradation	[102]
Ca-MOF	FDM	PLA	–	Detection of Hg(II)	[85]
Cu-BTC/ABS	FDM and seed assisted in situ growth	ABS	–	Dye adsorption	[54]
Ca-MOF/ABS/TPU	Coordination replication method	ABS/TPU	CaSiO_3_ (as a precursor of Ce-MOF)	Dye adsorption	[86]
UTSA-16(Co)	DIW	Bentonite clay	PVA (as a plasticizer)	CO_2_ adsorption	[55]
MOF-74(Ni)	DIW	Bentonite clay	PVA (as a plasticizer)	CO_2_ adsorption	[55]
UiO-66^b^	DIW	TMPPTA	Photoinitiator	Catalyst	[61]
HKUST-1^b^	DIW	Without using any binder	–	CH_4_ adsorption	[103]
Co-MOF	DIW	–	Pluronic F127 as a surfactant	Batteries	[62]
ZIF-8	DIW	Bentonite/methylcellulose	–	Recovery of n-butanol	[104]
MOF/CA-GE	DIW	SA-GE matrix	Ca(II) (as a crosslinking agent)	Dye adsorption	[94]
MOFs^c^	DIW	2-hydroxyethyl cellulose	PVA (as a plasticizer)	Gas storage and separation	[105]
MOF@torlon	Seed-assisted in situ growth	Torlon	PVP (as a pore former)	CO_2_ adsorption	[96]
Semiflex/ZIF-8/PVDF-HFP	FDM and seed assisted in situ growth	Semiflex	PVDF-HFP (as a pore former)	Just fabrication	[106]
HKUST-1	SLS	Nylon-12	–	Just fabrication	[107]
MOF/PA12^d^	SLS	PA12	–	Dye adsorption	[108]
MIL-53(Al)-NH_2_/MMA	DLP	Photopolymerizable oligomers	Photoinitiator	Gas separation	[65]
Cu-BTC@polymer	DLP	Photopolymerizable oligomers (e.g., SR-339 and SR-610)	Photoinitiator	Just fabrication	[66]

*PVP* poly(vinylpyrrolidone), *PVDF-HFP* poly(vinylidene fluoride-co-hexafluoropropylene), *MMA* methacrylic anhydride, *PLA* poly(lactic acid), *ABS* acrylonitrile butadiene styrene, *TPU* thermoplastic polyurethane, *PVA* poly(vinyl alcohol), *TMPPTA* trimethylolpropane propoxylate triacrylate, *PA12* polyamide 12, *SA-GE* gelatin and sodium alginate, *CA-GE* calcium alginate and gelatin

^a^Used as the rheology modifier

^b^MOF particles act as the rheological modifier

^c^CPL-1, UiO-66-NH_2_, ZIF-8, and HKUST-1

^d^MOF = MOF-801, ZIF-67, ZIF-8, HKUST-1, and NH_2_-MIL-101(Al)

It was reported that the chemically active MOF nanoparticles would be appropriate rheological modifiers. For instance, Young et al. [61] have demonstrated a UiO-66 ink with good printability, in which the MOF particles were employed as the rheological modifier without using further rheological modifiers like bentonite clay. Therein, the MOF nanoparticles in a solution containing acrylates and trimethylolpropane propoxylate triacrylate (TMPPTA) were 3D-printed via DIW strategy. After that, to increase the pore accessibility of MOF nanoparticles, the polymer matrix was selectively removed by thermal degradation. The fabricated 3D-printed MOF monoliths have a UiO-66 content of about 74 wt%, and a high BET surface area of 633 m^2^ g^−1^, which exhibited excellent activity in the catalytic degradation of methyl-paraoxon.

**Fig. 2:**
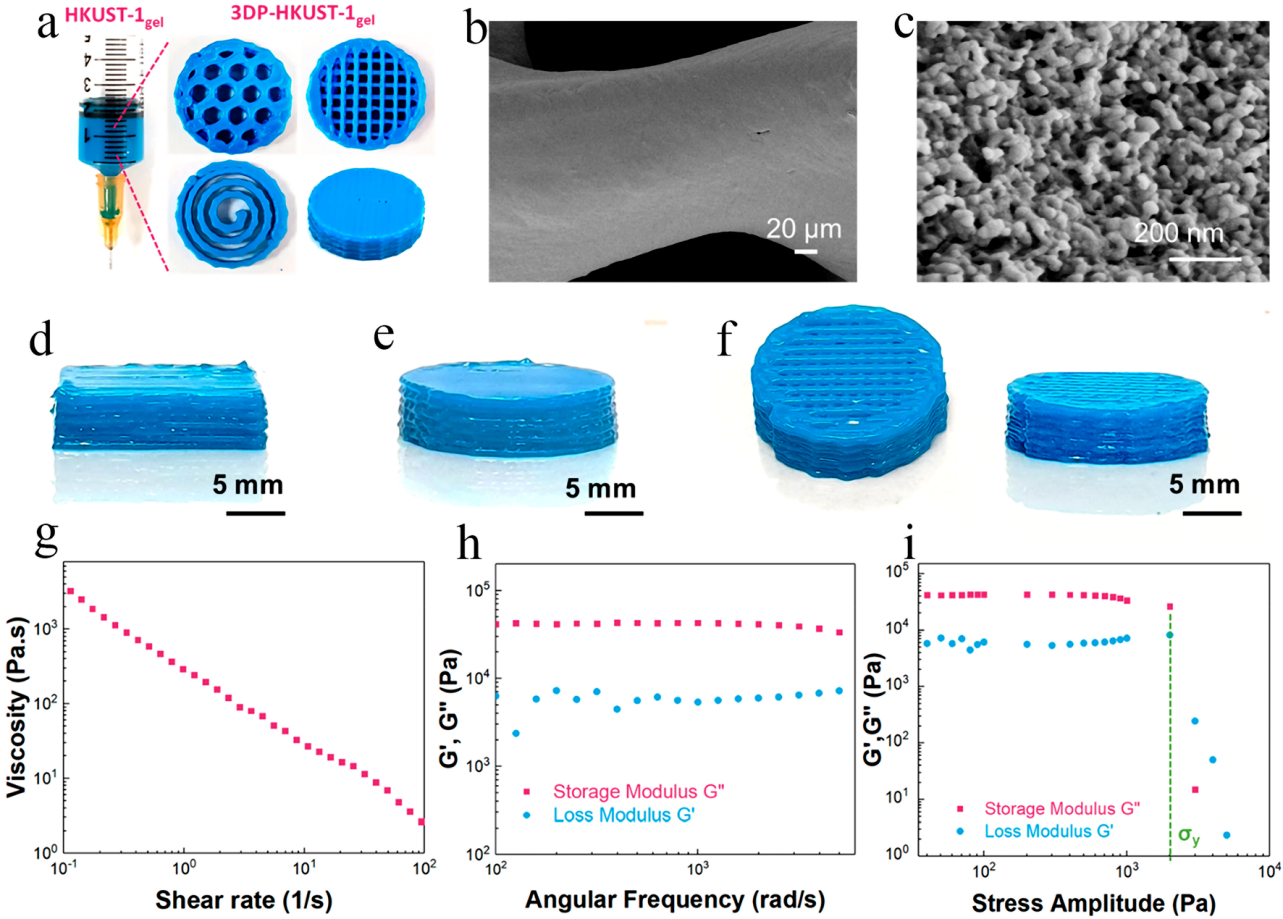
**a** Photo images of HKUST-1_gel_ and various 3D-printed monoliths. SEM images of **b** one filament of 3D-printed HKUST-1 monolith and **c** MOF nanoparticles within that. Photoimages of monolith with **d** square- and **e** circular-shape. **f** Side and top views of a monolith with a high-profile mesh-like structure. **g** Apparent viscosity profile against shear rate. **h** Storage modulus G′ and the loss modulus G″ as a function of angular frequency. **i** G′ and G″ as a function of shear stress-amplitude at a constant frequency of 6.283 rad s^−1^. Reprinted with permission from Ref. [103]. Copyright 2019, American Chemical Society

Similarly, Lim et al. [103] prepared a self-standing MOF monolith via DIW strategy, using colloidal gels containing only MOF (HKUST-1) nanoparticles and solvent (ethanol) without using any binder, then evaluated its methane adsorption performance performance (Fig. 2). The prepared MOF gel exhibited good printability (Fig. 2d–f), and excellent rheological features for 3D extrusion-based printing (Fig. 2g–i), indicating the applicability of this strategy for the fabrication of further monoliths that contain MOF particles and can form gels. More interestingly, it was reported that the pore volume and accessible porosity of the HKUST-1 nanoparticles were maintained unchanged after shaping. Accordingly, the 3D-printed HKUST-1 monolith showed a relatively high specific surface area (1134 m^2^ g^−1^), a high mesoporous volume, as well as a high methane uptake (64 cm^3^ (STP) cm^−3^ at 65 bar).

**Fig. 3:**
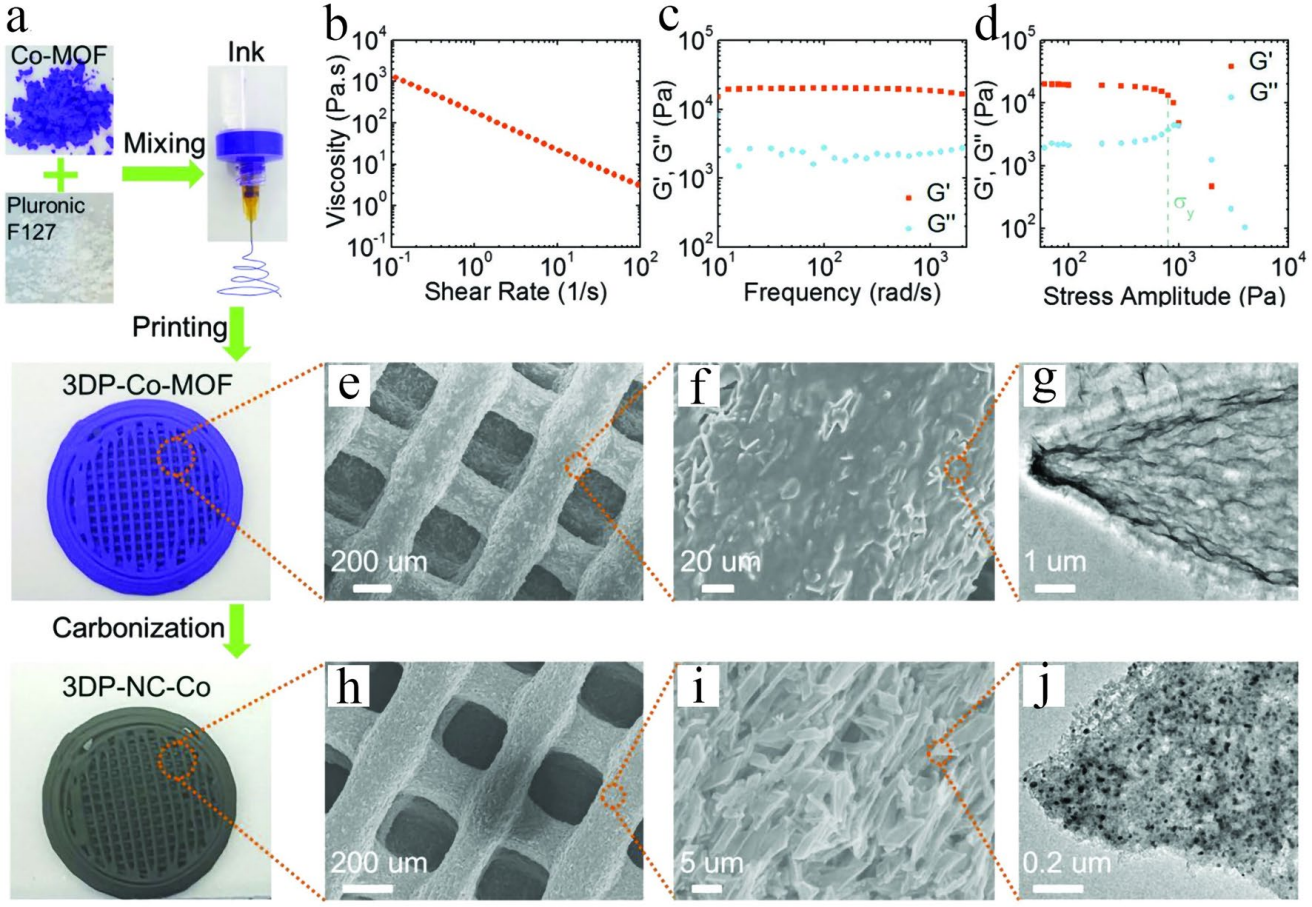
Fabrication and characterization of the 3D-printed Co-MOF-derived monolith. **a** Schematic representation of the fabrication processes. **b–d** Rheological behavior of the fabricated F127/Co-MOF ink exhibiting that the prepared ink is solid-like at rest and showed shear-thinning behavior by rising shear. **b** Apparent viscosity profile against shear rate. **c, d** Oscillatory frequency sweep results: **c** G′ and G″ profiles as a function of angular frequency. **d** G′ and G″ profiles as a function of stress-amplitude at a constant frequency of 6.28319 rad s^−1^. **e–g** Usual characterization of the fabricated 3D-printed Co-MOF. **e, f** SEM images. **g** TEM image. **h–j** Usual characterization of the fabricated 3D-printed NC-Co. Reprinted with permission from Ref. [62]. Copyright 2019, John Wiley & Sons

Lyu et al. [62] demonstrated the printing of 3D cobalt-based MOF (Co-MOF) monoliths from an ink containing a high amount of Pluronic F127 as a surfactant, which was further annealed to produce a Co-carbon porous framework to be used in Li–O_2_ batteries (Fig. 3). Figure 3a displays how of fabricating the designed cathode of a 3D-printed Co-MOF-derived structure via DIW strategy by employing an extrusion-based 3D printer. Accordingly, a suitable ink with good printability was prepared by dissolving Pluronic F127 and the Co-MOF nanoparticles in water at a low temperature (≈ 277 K) and poured into a syringe. Then, the prepared F127/Co-MOF ink was printed as high-resolution filament via layer-by-layer DIW to prepare the 3D-printed MOF monolith at ambient conditions, showing a gel-like behavior. Subsequently, the fabricated 3D-printed Co-MOF monolith was heated under nitrogen atmosphere, but the 3D structure was well maintained unchanged. As a result, the fabricated porous structure consisted of numerous micropores created between the MOF-derived carbon flakes and micro- and mesopores created within these flakes, which altogether remarkably improved the particle deposition of Li_2_O_2_ and facilitated their dissociation because of the restriction of insulating Li_2_O_2_ within the porous structure and in the existence of cobalt electrocatalysts. Thus, the fabricated self-standing structure with porous frameworks dramatically enhanced the cell’s practical specific energy, leading to high values (up to 798 Wh kg_cell_^−1^).

**Fig. 4:**
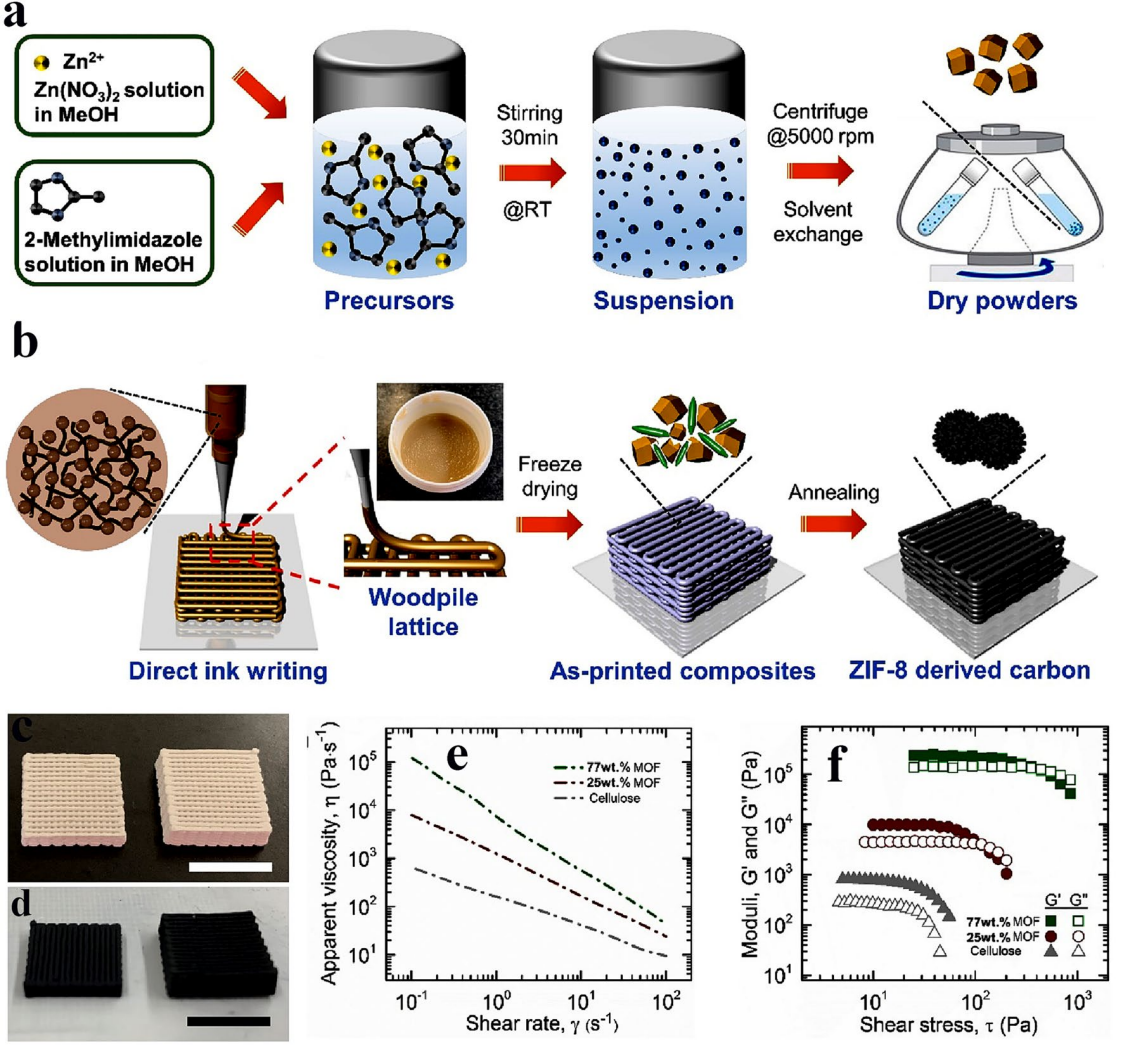
Schematic illustrating the preparation of **a** ZIF-8 particles and **b** ZIF-8 ink. Photoimages of **c** 3D-printed ZIF-8 and **d** pyrolyzed ZIF-8 (scale bar = 1cm). Rheological characterization results: **e** apparent viscosity and **f** loss and elastic modulus as a function of shear stress. Reprinted with permission from Ref. [109]. Copyright 2023, Elsevier

One of the challenges in the 3D-printing of MOFs with high loading via DIW is particle aggregation. Recently, Catarineu et al. [109] formulated an aqueous ink composed of cellulose nanocrystals (CNC) and ZIF-8 to produce a high-loading 3D-printed hydrogel (Fig. 4). They employed CNC as a surfactant to help with the deagglomeration of ZIF-8 particles. In addition, CNC acted as a binder and gelation agent to form hydrogen bonding with water molecules and hydroxyl groups of CNCs themselves in the hydrogel. The authors studied the influence of ZIF-8 loadings on the rheological properties, which is another characteristic decisive in the printability of the resulting ink. They realized that with increasing the ZIF-8 mass loading, the viscosity and internal stress increase, resulting in improved printability and preservation of the cohesion in the printed sample without the need for any rheological modifiers. Notably, the mass fraction of the MOF must be > 50 wt% to minimize shrinkage. Nevertheless, ZIF-8 loadings of > 77 wt% results in a dilatant solution, which is not extrudable because of the high yield throughout the flow. Thus, the 3D-printed sample with 77 wt% of ZIF-8 was introduced as the optimum specimen. The authors eventually pyrolyzed the printed ZIF-8 lattice to produce an electrically conductive and microporous structure with 660 m^2^ g^−1^ specific surface area to use as a hybrid supercapacitor cathodic electrode. Due to the high loading of ZIF-8, the 3D structure was well retained with minimal shrinkage (less than 10%) after the pyrolysis. The pyrolyzed sample consisted of micropores preserved from ZIF-8 template and mesopores formed in the graphitic carbon resulting from pyrolysis. As a result, the final fabricated zinc-ion hybrid supercapacitor demonstrated an areal capacitance of 16.9 F cm^−2^ caused by microporosity, high specific surface area, and good attractive interactions with ions present in the electrolyte thanks to the ZIF-8 template.

In other interesting work, Denayer and coworkers [104] prepared different types of 3D-printed ZIF-8 monoliths via DIW strategy employing either 600 or 250 µm diameter cells, then investigated their performances for dynamic recovery of n-butanol at different flow rates. It was found that among the two structured ZIF-8 monoliths, the smaller (250 µm) monoliths showed a wider breakthrough front in comparison to the larger one, largely because of the inappropriate distribution of the gas velocity throughout the width of smaller monolith channels, resulting in the inadequate exposure time for the adsorbate molecules to penetrate the pores of monoliths. Accordingly, the authors proposed that the 3D-printed ZIF-8 frameworks with larger porous and open structures are better for industrial n-butanol recovery applications.

**Fig. 5:**
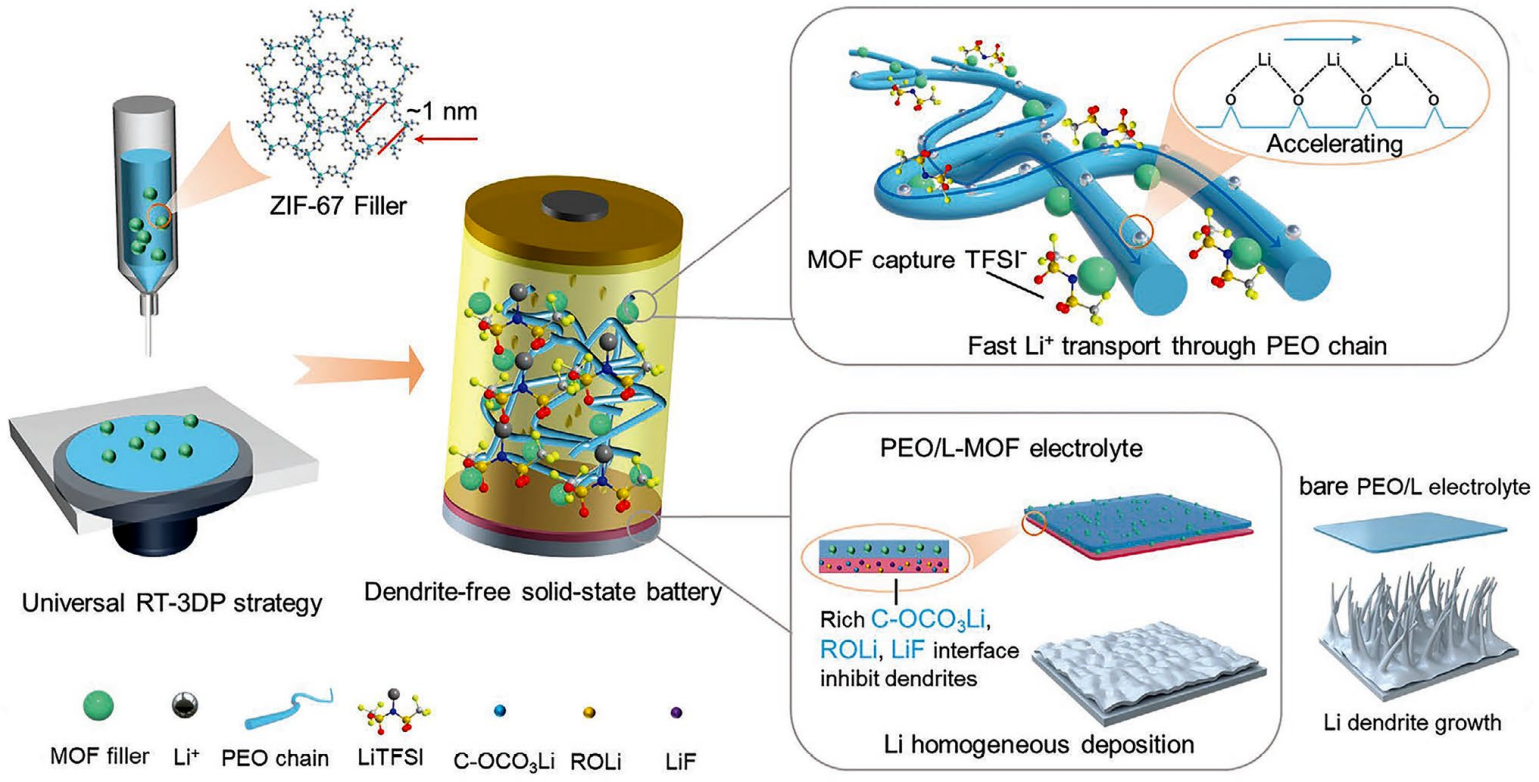
Schematic illustrating the preparation of hybrid solid-state electrolytes via the universal room-temperature DIW strategy towards dendrite-free solid-state lithium metal batteries. Reprinted with permission from Ref. [111]. Copyright 2023, John Wiley & Sons

Moreover, it was reported that the DIW strategy could produce favorable monoliths suitable for customization and modulation of components of hybrid solid-state electrolytes [110]. Inspired by this, Li et al. [111] prepared novel denderit-inhibited PEO/MOFs hybrid solid-state electrolytes through the room-temperature DIW strategy (Fig. 5). It was observed that incorporating MOF particles within the polyethylene oxide (PEO) matrix can significantly accelerate the transport of lithium ions (Li^+^), promote the homogeneity of lithium deposition, and improve the cyclic stability of resulting electrolytes via inhibiting the lithium dendrite. Meanwhile, pure PEO membranes don’t have the ability to inhibit dendrite growth, thereby leading to a shortened battery life. The authors believed that this strategy is a universal method that can produce several electrolytes containing different MOFs, including ZIF-8, ZIF-67, UiO-66, and MOF-74, promoting solid-state battery performances.

### Seed-Assisted In Situ Growth

Analogous the DIW strategy, 3D printing of monoliths fabricated via this strategy starts by condensing a printable ink, usually containing solvents, inert binders, and plasticizers. But, contrary to the DIW strategy, the structured monoliths fabricated by this strategy do not need a large amount of the active material to produce functionality. However, in this strategy, the 3D-printed substrate acts as a nucleation site that contains different metal oxides or MOF precursors (e.g., metal salts and organic ligands), which enables the coordinate of the active MOF layer after primary densifying [112]. As a result, the monoliths can be fabricated by secondary growth using chemical precursors and a template, and subsequently by in situ growth to prepare the chemically active samples [113].

This strategy has usually not led to relatively high MOF loadings or has resulted in the production of MOF phase with relatively poor crystallinity. For instance, Liu and coworkers [54] tried to synthesize Cu-BTC MOF onto the surface of 3D-printed ABS matrix and was impotent to produce a 3D-printed MOF monolith with MOF content of more than 15 wt%. Similarly, Lawson et al. [96] demonstrated the interfacial synthesis of MOF-74 onto the surface of 3D-printed MOF-74/torlon monoliths. To promote the phase separation process and produce solid monoliths, the MOF nanoparticles were 3D-printed into a dope of torlon solution in contact with a non-solvent. However, the crystalline structure of the MOF-74 nanoparticles was degraded during the solvent extraction process. But, when these MOF-74/torlon monoliths were exposed to the MOF precursors, the collapsed MOF-74 particles successfully acted as growth seed, resulting in the fabrication of monoliths with 40 wt% MOF-74 loading. Although this strategy could produce the active monoliths, it is relatively time consuming and leads to the production of extra waste, demonstrating that this strategy is not very suitable for scale-up.

Integrating the benefits of the 3D-printing technology with the favorable properties of the seed assisted growth strategy, encouraged Lawson et al. [114] to prepare 3D-printed MOF structure, which enhance MOF content, mechanical strength, and gas adsorption performances. Therein, for fabrication of 3D-printed MOF monolith, at first, the pastes containing metallic precursor and inert substrate were printed. The related MOFs were synthesized by transforming the incorporated metallic precursor into MOFs via a secondary solvothermal synthesis technique. Accordingly, the 3D-printed monoliths containing zeolite 13X, bentonite clay, mesoporous silica, and kaolin synthesized the related MOFs. However, this strategy was found to be only efficient for producing UTSA-16(Co) using 3D-printed kaolin monolith. As a result, the fabricated MOF-based monolith with 90 wt% UTSA-16(Co) loading showed the same physical properties and gas adsorption performances as its powder form whereas upgrading its structural integrity. The fabricated 3D-printed UTSA-16-kaolin structure showed a nearly similar CO_2_ uptake to the MOF powder (3.1 vs 3.5 mmol g^−1^ at room temperature and 1 bar), related to the amount of MOF loading. Moreover, this adsorbent exhibited excellent separation selectivity of 3725, 238, and 49 toward CO_2_/H_2_, CO_2_/N_2_, and CO_2_/CH_4_, respectively, indicating exceptional separation performances of the 3D-printed MOF monoliths for different mixtures of gases.

**Fig. 6:**
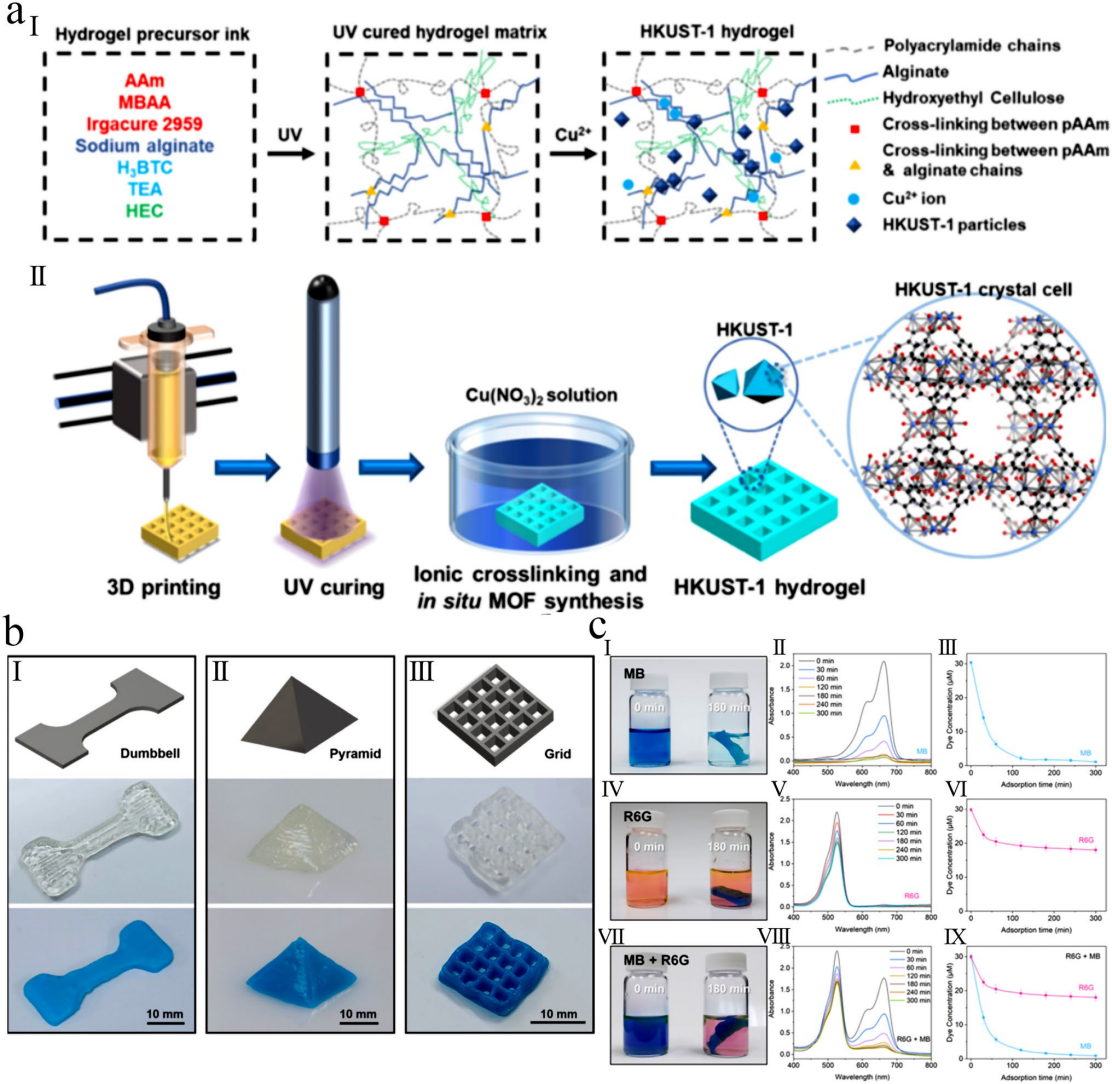
**a** Schematic representation for the composition and 3D printing procedure for fabrication of MOF hydrogels. (I) Chemical composition of precursors of various phases: (left) hydrogel ink, (middle) UV-cured hydrogel phase, and (right) the MOF hydrogel. (II) Schematic illustration of the three main stages (printing, UV curing, and ionic cross-linking) in the 3D printing process. **b** Fabricated 3D-printed HKUST-1 hydrogel composites in various forms. (I-III) (top) 3D rendering, (middle) photoimages of 3D-printed hydrogel phase, and (bottom) 3D-printed MOF hydrogel composites in various forms printed by a nozzle with a diameter of 0.8 mm. (I) dumbbell, (II) pyramid, and (III) grid. **c** Organic dye adsorption efficiency of the fabricated 3D-printed MOF hydrogels. Photoimages for different organic dye solutions at the beginning and after the adsorption process for (I) MB, (IV) R6G, and (VII) a mixture of MB and R6G dyes. The UV–Vis spectra of (II) MB, (V) R6G, and (VIII) MB + R6G solutions as a function of contact time. The concentration of various dyes in (III) MB, (VI) R6G, and (IX) MB + R6G mixture as a function of contact time. Reprinted with permission from Ref. [115]. Copyright 2020, American Chemical Society

In another study, Liu et al. [115] demonstrated a novel 3D printing strategy to prepare a tough and stretchable MOF-hydrogel composite with adjustable mechanical characteristics. As shown in Fig. 6a, they formulated an efficient printable ink by mixing pre-polymers of a flexible double network (DN) hydrogel of alginate and acrylamide, the organic linkers of MOF, and a shear-thinning agent. Therefore, due to concurrent cross-linking of alginate and in situ synthesis of HKUST-1 crystals onto the surface of hydrogel using copper ions, a composite with high MOF dispersity and high pore accessibility would be prepared. The fabricated 3D-printed HKUST-1 hydrogel was shaped in various morphologies for further characterization (Fig. 6b). They also investigated the adsorption performance of this MOF hydrogel for organic dye removal, which showed excellent performance of the MOF hydrogel for selective dye adsorption (Fig. 6c). Similarly, Huang and Wu [116] demonstrated that by immersing a 3D-printed skeleton containing organic ligands in a solution containing the related metal ions, the in situ synthesis of Ln-MOFs can quickly occur, leading to macroscopic assemblies and adjustable fluorescence abilities.

A challenge in 3D printing MOFs is their limited compatibility with binders, leading to unfavorable rheological properties. To increase the interfacial interaction between MOF nanoparticles and binder, an MOF construction was formulated by Rezaei and coworkers [117] through 3D printing of gelated chemical precursors and their in situ synthesis. To overcome the MOFs’ rheological limitations, they developed a new fabrication strategy named gel-print-grow (GPG), in which the 3D-printed monolith was printed by DIW strategy. In this novel strategy, the as-prepared sol–gel should have the following properties: (i) show low spreading behavior, (ii) exhibit a suitable self-standing rheology, and (iii) be stable under operating conditions to guarantee the complete coordination of the crystalline phase. Thus, to successfully fabricate a 3D-printed monolith via this strategy, selecting gelation agents and secondary MOFs growth conditions is very important.

**Fig. 7:**
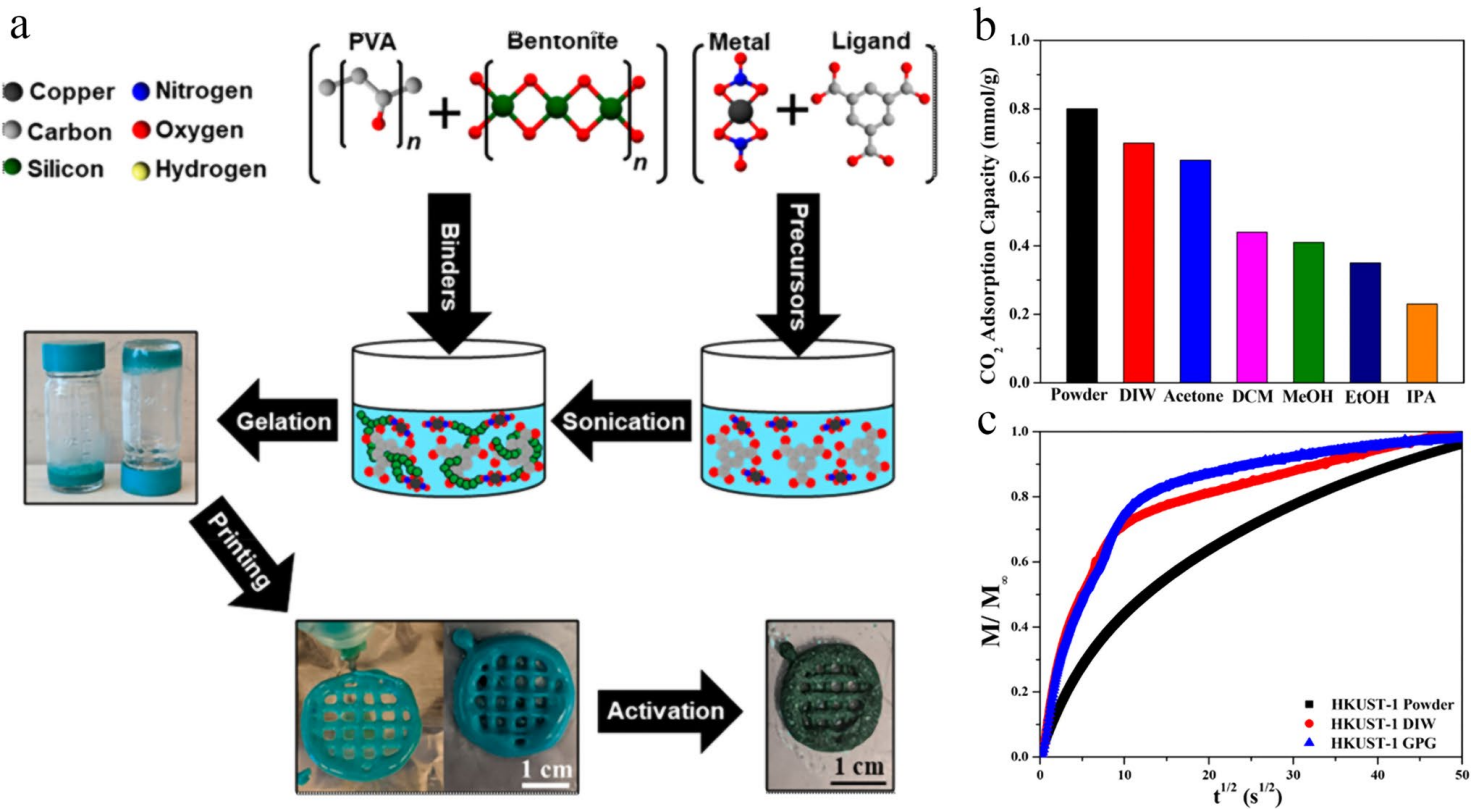
**a** Schematic illustration of HKUST-1 monoliths prepared by gel-print-grow strategy. **b** CO_2_ adsorption capacities of all synthesized samples at room temperature and 1 bar as a function of activation solvents. **c** Fractional adsorption capacities of CO_2_ in a CO_2_/N_2_ (10:90) mixture at room temperature for HKUST-1 powder and its monoliths fabricated via different strategies. Reprinted with permission from Ref. [117]. Copyright 2020, American Chemical Society

As shown in Fig. 7a, for the synthesis of a sol–gel with good printability, a mixture of related MOF precursors was produced, followed by sonication, then poly(vinyl alcohol) (PVA) and bentonite clay were added into it, which resulted in the formation of the gel. Usually, the bentonite clay can improve the rheological characteristics (self-standing rheology) of the prepared ink. As a result, they synthesized a sol–gel with good printability that contains approximately 70 wt% of MOF precursors and optimized the in situ synthesis situations by altering the temperature of desolvation and activation solvents. Accordingly, it was found that the desolvation temperature played a critical role in the synthesis of MOF crystals, as the HKUST-1 crystals were completely formed at 393 K. Additionally, the activation solvent led to the differences in the textural properties of the fabricated monoliths in which using solvents with lower volatility like ethanol or 2-propanol may result in the formation of a structure with smaller pore space. While utilizing solvents with higher volatility, including methanol and acetone, may result in higher CO_2_ uptakes at room temperature and 1 bar (Fig. 7b). However, among acetone and methanol solvents, methanol resulted in the decomposition of crystalline structure of HKUST-1 particles, so acetone was considered superior solvent for activating this MOF. More importantly, the MOF monoliths fabricated through the sol–gel printing and coordination strategy usually exhibited quicker mass transfer characteristics in comparison to an equivalent 3D-printed MOF monolith prepared by DIW strategy, thanks to the formation of extra pores in the gelated samples, thereby enhancing the molecular transportation.

**Fig. 8:**
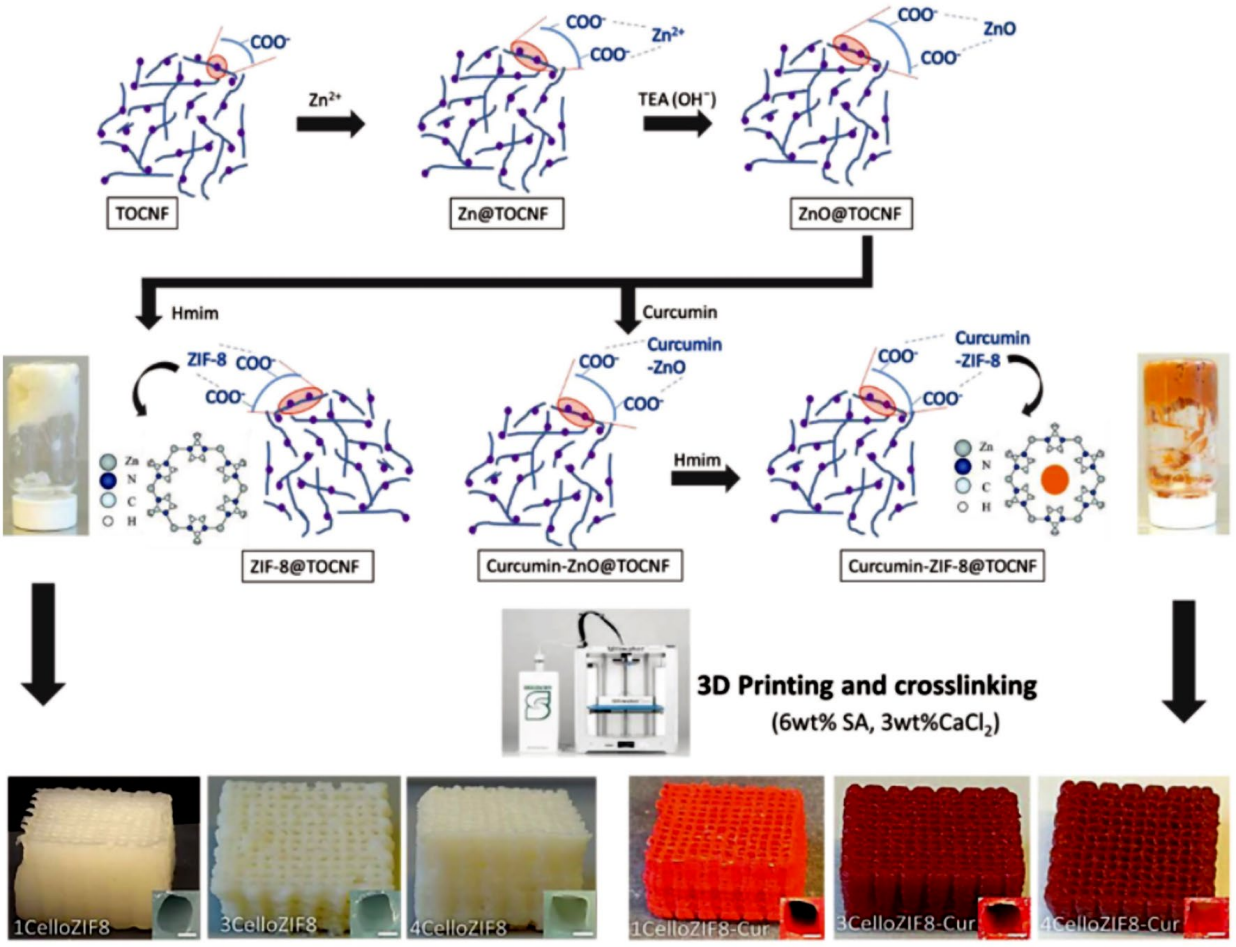
Schematic representation for the preparation and 3D printing of CelloZIF8 composite inks with and without adding curcumin molecules. The printed monoliths with orange color exhibit the existence of curcumin molecules in the prepared composite ink. Reprinted with permission from Ref. [120]. Copyright 2019, John Wiley & Sons

Recently, the formation of nanocomposite inks that utilize nanocellulose networks to encapsulate various nanoparticles and functional materials has attracted great attention [118]. For this goal, post-treatments, like cross-linking are usually applied to improve the mechanical integrity or fulfill the requirements of the application [119]. For example, Sultan et al. [120] demonstrated a one-step synthesis of 3D printable hydrogel ink composed of ZIF-8 nanoparticles attached to anionic 2,2,6,6-tetramethylpiperidine-1-oxylradical-mediated oxidized cellulose nanofibers (TOCNF) (Fig. 8). Therein, Zn^2+^ ions were dispersed into the TOCNF solution under vigorous stirring, which coordinated to the –COOH groups of nanofibers and consequently controlled MOF crystal growth [121]. Additionally, triethylamine (TEA), as an efficient nucleation agent, was dissolved into the solution, which led to the creation of ZnO nanoparticles. After the addition of the related organic ligands (Hmim = 2-methylimidazole), the crystals of ZIF-8 would be formed due to the successful conversion of ZnO nanoparticles into the ZIF-8 nanoparticles. Accordingly, they believed that the synthesis strategy of ZIF-8@TOCNF (CelloZIF8) hybrid ink is facile, rapid, environmentally friendly (using water as a green solvent), usually takes place at ambient conditions, and allow facile encapsulation of small molecules like methylene blue (MB) and curcumin. The shear-thinning behavior of the fabricated hydrogel composite inks facilitated the 3D printing of porous monoliths with exceptional shape loyalty, in which the final monolith showed pH controlled release of encapsulated curcumin molecules [120].

**Fig. 9:**
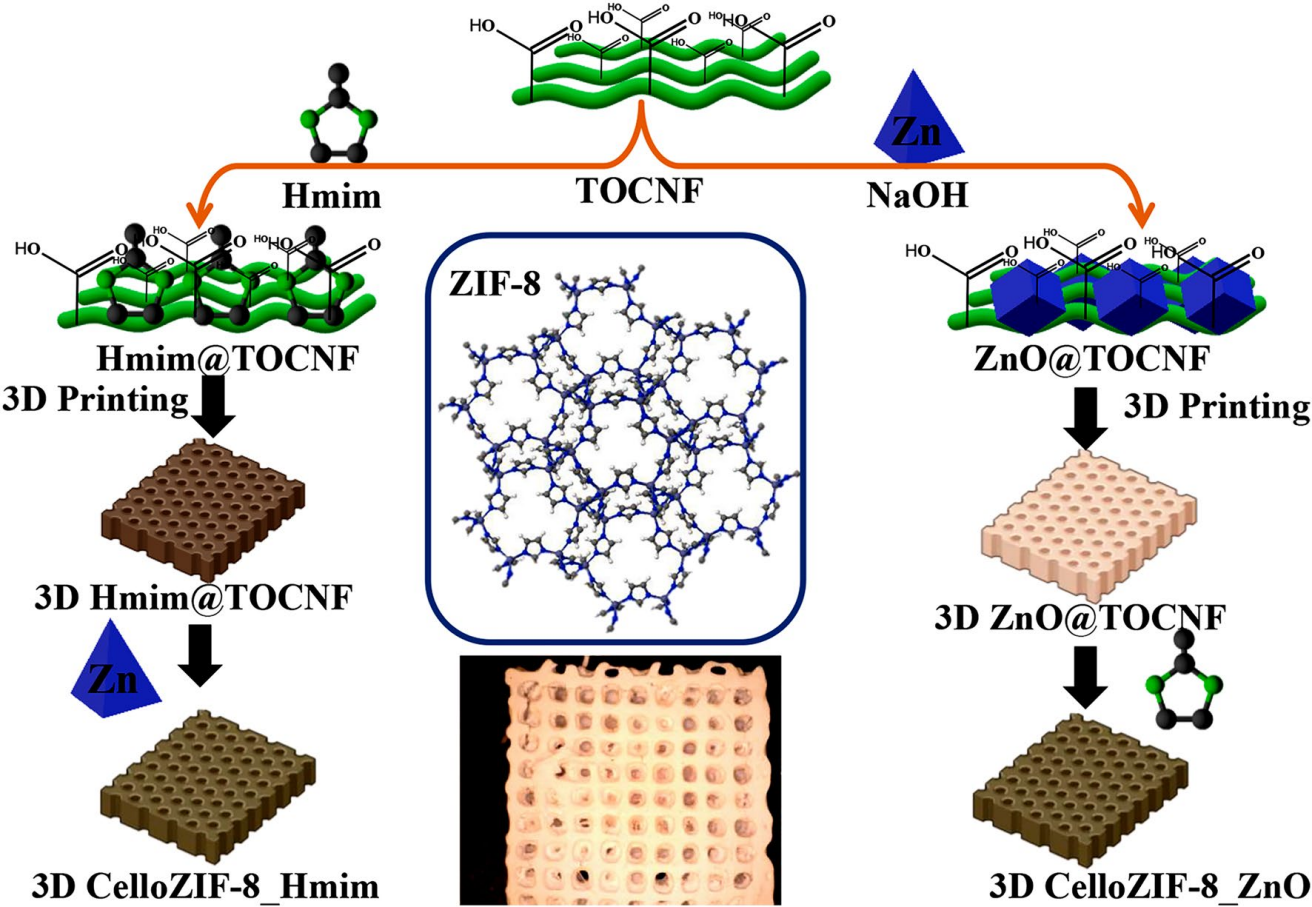
Schematic illustrating the one-pot preparation of 3D CelloZIF-8 monolith. Reprinted with permission from Ref. [122]. Copyright 2023, Elsevier

The same research group developed a similar approach for preparing a Cellulose-ZIF-8 (CelloZIF-8) hydrogel. In this regard, Abdelhamid et al. [122] introduced Hmim into an aqueous solution containing TOCNF to create a printable ink (Fig. 9). The authors mentioned that, besides serving as the precursor for ZIF-8 growth, Hmim also acted as a suitable gelation agent for TOCNF. Following the 3D printing of the ink, the resulting object was immersed in a saturated solution containing Zn^2+^ to trigger the in situ growth of ZIF-8 particles. Hmim molecules interacted with the carboxylic groups of TOCNF through coordination or hydrogen bonding. This process paved the way for the creation of stable 3D objects without the use of any binders. Nevertheless, XRD analysis revealed a hydrolyzed form of ZIF-8 particles, indicating that this method could not yield completely pure ZIF-8 crystals. Morphological studies showed a porous structure with pores measuring approximately 1 mm. ZIF-8 particles were uniformly distributed within the CelloZIF-8 structure, with sizes ranging from 100 to 250 nm. CelloZIF-8 was employed in various applications, including CO_2_ adsorption, heavy metal adsorption, and the removal of organic dyes through adsorption and catalytic degradation.

In a recent study, Chen et al. [123] reported the development of a multifunctional hydrogel containing cerium-based MOF for treating and monitoring diabetic wounds. The hydrogel comprised a dual network of Ce-MOF connected to a sodium alginate network and polyacrylamide (PAAm), with these two networks forming an interpenetrating polymeric network. To prepare the bioink, the authors introduced a solution of TEA and the organic linker to the polymer mixture, neutralizing the carboxylic acid groups of the organic linker via TEA to achieve ligand dehydrogenation at room temperature. UV curing was employed during 3D printing and subsequent formation of the 3D structure to ensure the polymerization of acrylamide and promote cross-linking. The prepared hydrogel was immersed in a cerium nitrate solution for dual purposes. Firstly, it resulted in the formation of crosslinked sodium alginate with Ce(III). Secondly, excess Ce(III) ions reacted with the linker, generating Ce-MOF within the porous network of the hydrogel. XRD analysis demonstrated that the crystal structure of the grown MOF resembled its powdered form. Morphological studies revealed that the prepared scaffold fully retained its porous structure. Additionally, it was observed that Ce-MOF exhibited a cauliflower-like morphology, which was attributed to the split growth of the MOF during crystal formation. Ce-MOF improved the mechanical properties of the hydrogel, providing physical protection for the wound. The fabricated MOF nanozyme hydrogel exhibited remarkable catalytic activity towards various oxygen-free radicals (providing reactive oxygen species (ROS) scavenging properties). It showed dependency on the glucose concentration, owing to the conversion between different valences of cerium.

### Coordination Replication from Solid Precursors

MOF growth from solid precursors (e.g., metal oxides) via coordination replication strategy has been explored as a simple and efficient approach to deposit MOF coating on substrates [124]. The conversion of zinc oxide (ZnO) layer to ZIF-8 crystals under mild conditions has been achieved in various supporting materials including fibers, beads, and monoliths [125]. For instance, by FDM strategy, Waheed et al. [126] prepared a chemically reactive ZnO nanoparticle (NP)/ABS composite filament for 3D printing. After 3D printing with the ZnO-NP/ABS filament, the ZIF-8 particles were in situ synthesized via a moderate chemical conversion mechanism at ambient conditions, in which the incorporated ZnO-NP converted into the ZIF-8 particles that were consequently located on the external surface of 3D-printed monoliths. The fabricated 3D-printed MOF monolith was used to extract malachite green (MG) from aqueous solutions, which showed exceptional extraction performance compared to a monolith prepared from net ABS, or the fabricated ZnO-NP/ABS monoliths. To better understand the benefit of this strategy for 3D-printed monolith fabrication, a ZIF-8/ABS monolith was prepared by directly incorporating ZIF-8 nanoparticles within the ABS matrix. As a result, the in situ growth of ZIF-8 nanoparticles via coordination replication strategy exhibited enhanced extraction performance compared to the monoliths containing pre-synthesized ZIF-8 nanoparticles, with a high enhancement (up to 48%) in the extraction of MG.

The surface modification of 3D-printed monoliths with MOF particles usually requires an efficient substrate seeding with nanocrystals of various MOFs [127]. However, uniform dispersion of seeds within the complex 3D structures is usually challenging; thereby, it is difficult to uniformly grow a layer of MOF crystals on the external surface of a substrate. In this regard, Pellejero et al. [128] reported a new coordination replication strategy based on reactive substrate seeding that involves the deposition of metallic (metal oxides) precursors by atomic layer deposition (ALD) [129], and its solvothermal conversion to the related MOFs. According to this synthesis strategy, ALD is able to functionalize the ABS membranes with a homogeneous layer of ZnO crystals, thereby producing a uniform distribution of metallic precursor for uniformly growing the crystals of ZIF-8 on the surface of ABS matrix. The SEM images of fabricated monoliths showed that the homogeneous and continuous layer of ZIF-8 nanoparticles was present in the inner surface of the 3D-printed ABS/ZIF-8 monoliths. The fabricated 3D-printed ABS/ZIF-8 monoliths showed an encouraging adsorption performance for dimethyl methylphosphonate (≈ 20.4 mg g^−1^), as a famous G-series nerve agent simulant, indicating their great potential for toxic gas removal applications.

Although directly growing MOF particles onto the external surface of 3D-printed frameworks appears to be a possible alternative strategy, most of the fabricated 3D-printed MOF-based monoliths were derived from Cu, Zn, and Co metals and possess different disadvantages because of the complex operation, long reaction time, and additional contamination produced by the metal ion solutions. Lately, calcium-based salts have been used as metallic precursor to synthesize MOFs due to their low-cost, nonpoisonous, abundant, and suitable bio-melting characteristics [130]. For instance, Kitagawa and coworkers [131] used calcium carbonate (CaCO_3_) as a metallic precursor and transformed it into a Ca-based MOF via the coordination replication. Based on this strategy, Liu et al. [86] fabricated a 3D-printed MOF-based monolith to efficiently remove MB dye from wastewater. Therein, CaSiO_3_, as a metallic precursor, was incorporated into the ABS/TPU alloy using a twin-screw extruder, and then the CaSiO_3_/ABS/TPU filaments were extruded on a mini filament structure for further 3D printing process. The Ca-based MOF crystals were directly deposited onto the external surface of the acetone-etched 3D structure via an in situ MOF synthesis technique. The fabricated 3D Ca-MOF/ABS/TPU structure showed good adsorption performance toward MB removal, in which its removal efficiency reached 88% for 100 ppm MB solution. Additionally, the removal efficiency was still at 70% after six consecutive adsorption–desorption cycles, indicating the high MB removal efficiency as well as good reusability of Ca-MOF/ABS/TPU structure.

### Matrix Incorporation

**Fig. 10:**
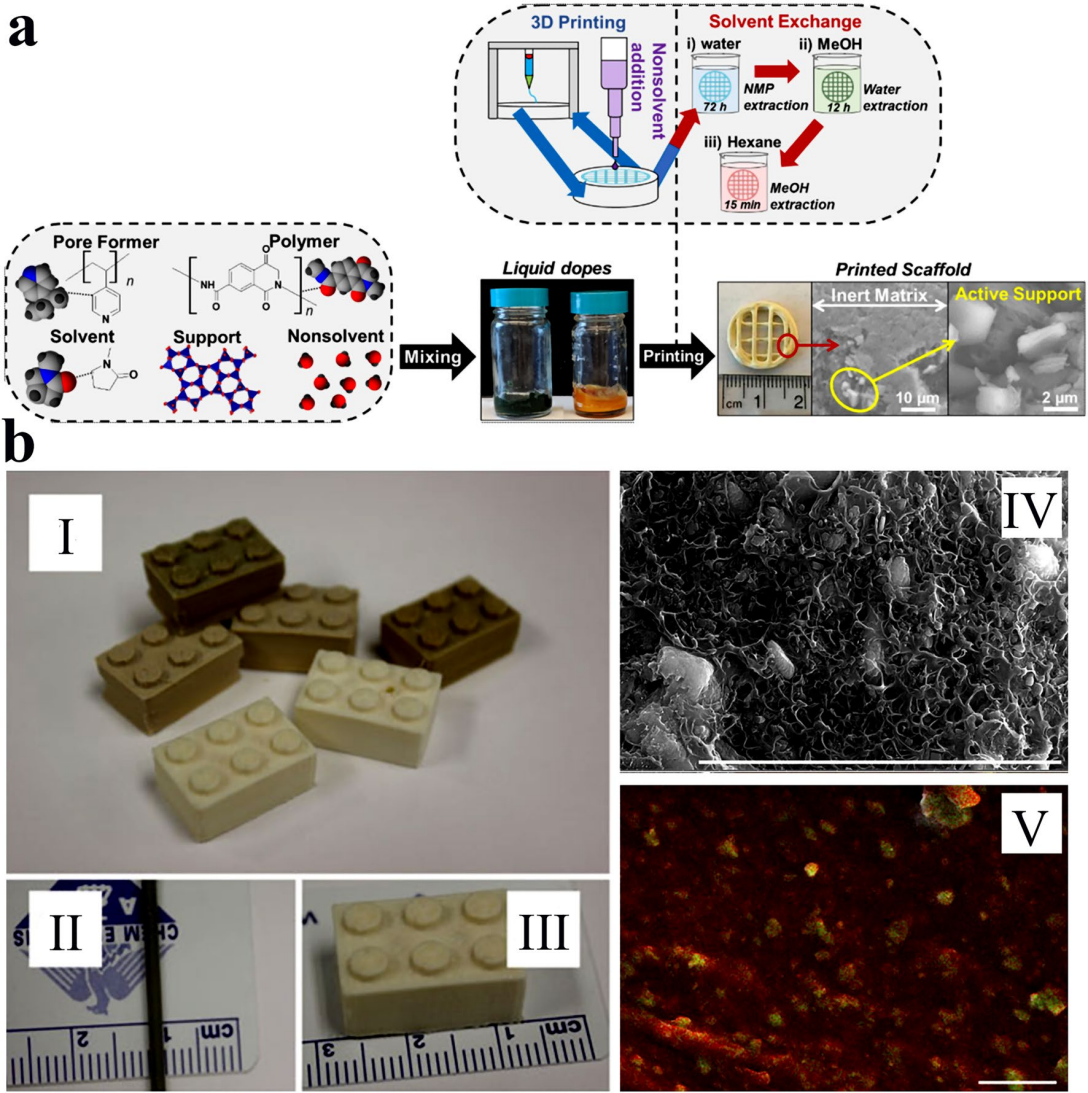
**a** Schematic representation for the 3D-printed MOF monolith formulation via the matrix incorporation strategy. Reprinted with permission from Ref. [113]. Copyright 2021, American Chemical Society. **b** Photoimages of the ABS-MOF-5 filament and the 3D-printed structures: (I) 3D-printed block prepared with various MOF-5 loadings. (II) Photo image of the filament utilized in the printing process. (III) 3D-printed structure shows scale with corresponding (IV) SEM and (V) zinc maps. Reprinted with permission from Ref. [59]. Copyright 2018, John Wiley & Sons

Matrix incorporation strategy is simply explained as the dispersion of chemically active materials (e.g., polymeric matrix and filler particles) within an inert stabilizer [132]. This fabrication strategy has usually been applied to overcome the rheological limitations and upgrade the mechanical properties of the 3D-printed frameworks or to modulate their hierarchical porosity and the accessibility to their active sites. As shown in Fig. 10a, for preparation of 3D-printed MOF structures via this strategy, the pre-synthesized MOF particles were firstly dispersed into a mixture containing polymer, solvent, pore former, and non-solvent to produce a liquid-phase dope with a desirable printability and rheology. Subsequently, a non-solvent is added on the printed layer to promote phase separation and form a solid phase. These processes are repeated several times until the favorable height monolith has been fabricated. Finally, the solvents are extracted from the fabricated 3D-printed MOF monoliths in exchange for increasingly volatile solvents [96, 133].

Kreider et al. [59] used this strategy for the first time to prepare 3D-printed ABS-MOF-5 composites with different geometries (Fig. 10b) by the solvent casting of an ABS mixture containing 10 wt% of MOF-5 particles. The obtained result for energy dispersive X-ray spectroscopy (EDS) analysis affirms the uniform dispersion of zinc as well as MOF-5 particles within the ABS matrix (Fig. 10b(V)). The authors believed that their fabrication strategy was an efficient and successful technique for fabricating MOF monoliths via simple solvent casting. However, they found that the MOF content cannot be enhanced by more than 10 wt% because the fabricated ink above that threshold showed shear thickening behavior and could no longer be printed. Thus, the proposed strategy would not be a successful and effective strategy for structuring MOF materials with high particle loadings, so it is essential to develop new alternatives to overcome this challenge.

With a similar strategy, Lawson et al. [96] prepared a series of 3D-printed MOF@torlon monoliths via suspending MOF nanoparticles (e.g., HKUST-1 and MOF-74) into a polymer solution and employing a phase separation process to prepare the favorable MOF monoliths. This effort was carried out to significantly improve the rheological properties of directly printed MOF monoliths because the liquid mixture of polymers shows better printability and shear-thinning behavior than the MOF inks prepared from DIW strategy. Therein, HKUST-1 and MOF-74 monoliths, with a polymer composition of 60 wt% polyamide (imide) (Torlon) and 40 wt% MOFs, were printed and investigated their adsorption performances toward CO_2_. It was found that only HKUST-1 monoliths showed complete crystalline preservation of MOF particles, while the polymer solvent slightly decomposed MOF-74. However, the preserved MOF particles were further employed as growth seeds to produce MOF-74-decorated monoliths. Accordingly, it was found that the secondary growth of MOF-74 particles on the external surface of monoliths significantly increased the CO_2_ uptake of MOF-74@torlon. Based on the results, the authors proposed that the direct 3D printing of MOF precursors that act as seeds and the use of secondary MOF crystal growth are suitable and attractive strategies for manufacturing polymer-based MOF monoliths. However, despite several advantages of this strategy, it is not optimal because it does not produce MOF ink with favorable rheological behavior, which may lead to the decomposition of the MOF crystals. Therefore, this strategy should be further developed to overcome the abovementioned challenges.

In an interesting study, Evans et al. [106] proposed that a 3D-printed MOF monolith fabrication via a simple solution blending method and then casting into solid phases prior to extrusion into printing monoliths is very important to attain a continuous and homogeneous dispersion of MOF particles even at high MOF contents (50 wt%). In that study, ZIF-8 nanoparticles were homogeneously incorporated into PLA and TPU matrices at high MOF loadings (more than 50 wt%), then extruded into solid filaments, and finally utilized to form 3D-printed ZIF-8 monoliths by FDM strategy. The fabricated 3D-printed PLA/ZIF-8 monoliths showed a large specific surface area of 531 m^2^ g^−1^ and a hierarchical porous structure, in which its hierarchical porosity originated from a combination of the retained crystalline structure of ZIF-8 particles, micro- and mesopores, and huge micro-voids in the structure of fabricated composites that were observed at 40 wt% ZIF-8 loadings.

**Fig. 11:**
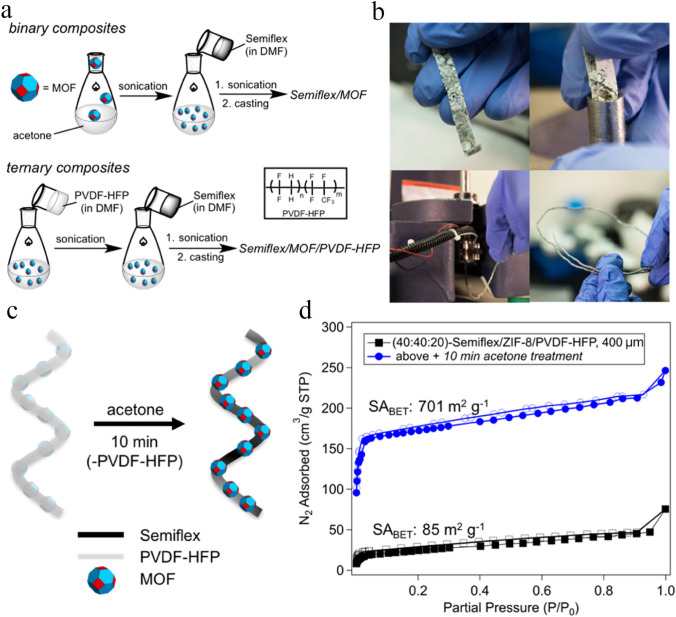
**a** Schematic illustration of the solution processes to produce binary (Semiflex) and ternary mixtures. **b** Semiflex-ZIF-8 composite monoliths cut, fed into a heated barrel, compressed to prepare extrusion, and produced feedstock materials via extrusion. **c** Schematic illustration of the flexible ternary ZIF-8 composite after 3D printing where PVDF-HFP blockages the porous structures of ZIF-8 particles, then acetone treatment removes PVDF-HFP to enhance the exposure of the incorporated ZIF-8 nanoparticles, while Semiflex is maintained as the foundation of polymer monolith. **d** Nitrogen adsorption–desorption isotherms of ternary Semiflex/ZIF-8/ PVDF-HFP 400 µm stands as printed and after solvent leaching. Reprinted with permission from Ref. [106]. Copyright 2018, American Chemical Society

In the case of flexible TPU/ZIF-8 composites, the huge voids are disappeared, and micropores are usually blockaged at a relatively high loading of MOF particles. Accordingly, the printed binary composite of Semiflex/ZIF-8 (50 wt% ZIF-8) showed a low specific surface area of 68 m^2^ g^−1^, slightly higher than that of monolith prepared from pure Semiflex. Therefore, the fabrication of flexible TPU/ZIF-8 composites with higher surface area was achieved by a new strategy in a sacrificial fluoropolymer (poly(vinylidene fluoride-co-hexafluoropropylene) (PVDF-HFP)) was doped in, preserved throughout the printing process, and finally separate from the fabricated monoliths via chemical treatment (Fig. 11c). Accordingly, to further enhance the specific surface area of the 3D-printed MOF composites and exposure of the pores within the incorporated ZIF-8 particles in the flexible Semiflex matrix, PVDF-HFP was employed to produce a ternary mixture (Fig. 11a). The PVDF-HFP was completely preserved in every part of processing and printing and subsequently eliminated from the composite via solvent treatment method to fabricate internal pores without changing the macrostructure of the final composite (Fig. 11c). As a result, the fabricated 3D-printed flexible Semiflex/ZIF-8 composites achieved a high surface area of 706 m^2^ g^−1^ (Fig. 11d), much more than that of the printed binary composite. This fabrication strategy was also extended to other MOFs (e.g., UiO-66) to exhibit the universality of this technique for fabricating highly porous MOF monoliths. However, the use of polymeric additives as sacrificial agents as well as using chemically active fillers to enhance the porosity, is an approximately unexplored field, which needs further investigation in the future.

### Selective Laser Sintering

Selective laser sintering (SLS) is the other critical 3D-printing strategy, in which the 3D solid phase is usually manufactured via the layer-by-layer sintering of hot-melt powdered materials (e.g., ceramics, metals, and thermoplastic polymers) utilizing a program-controlled laser beam [134]. During this fabrication strategy, the powdered precursors are slightly sintered together, by which the degree of this phenomenon could be controlled by accurately adjusting the printing factors, including exposure time, laser power, cooling rate, and operating temperature in the printing process, thereby regulating the physical characteristics of the fabricated monoliths like porosity [135]. As the operating conditions of the printing process are exactly optimized, the polymeric powders are not fully melted after the sintering process but still maintain their particle-like structure, which resulted in the fabrication of a porous, solid, and powder-like monolith that contains facile accessible spaces between the partially sintered polymer powders [136].

During the SLS process, micro-voids might be created as the powdered particles melt or sinter quickly using transient laser heating. When MOF nanoparticles and polymer powders are mixed for SLS process, the created micro-voids can produce open and free channels to the MOF nanoparticles incorporated in the polymer phase and significantly improve the exposure of MOF nanoparticles with the external ambient. Accordingly, the size and number of these micro-voids could be controlled by fine-tuning the sintering factors such as scanning speed, power, and hatching space of the laser. As a result, this relatively porous architecture permits liquids to pass through the fabricated monoliths even if no certain open channels are embedded in the porous structure of monoliths. In this regard, Lahtinen et al. [107] used SLS strategy to print highly porous membranes containing HKUST-1 as highly porous filler and nylon-12 as printable polymer phase. It was found that the MOF nanoparticles were tightly connected to the external surface of partially fused polymer powders, allowing liquids to pass through the membranes. Finally, the authors believed that this work displays that SLS strategy can open up a new window to use MOF nanoparticles by attaching them to an adjustable polymeric matrix.

**Fig. 12:**
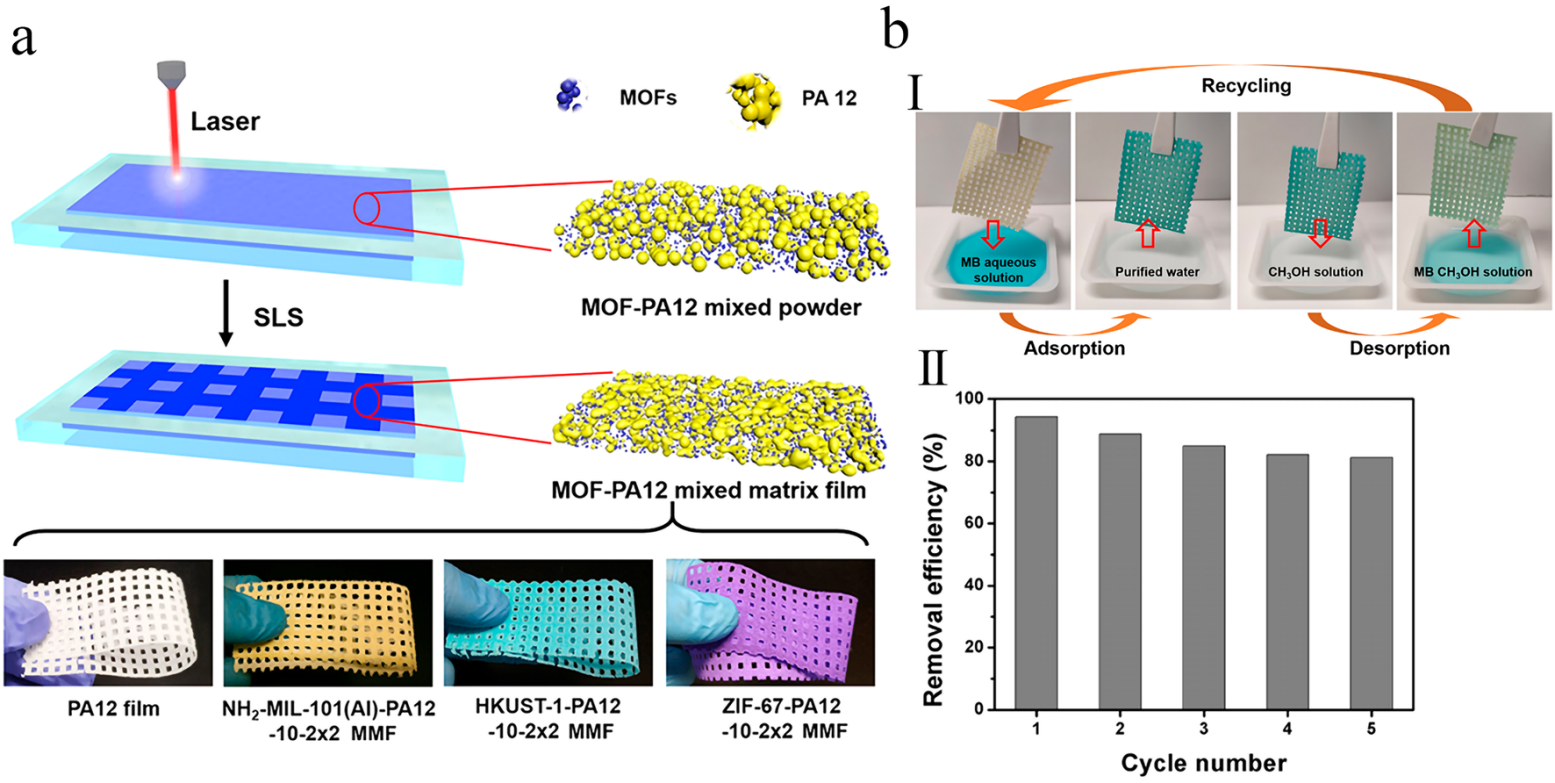
**a** Schematic representation for the manufacturing processes of 3D-printed MOF-PA12 MMFs with different lacing structure and photoimages of the PA12 film and MOF-PA12 MMFs containing different MOFs. **b** Recycling experiment of the NH_2_-MIL-101(Al)-PA12-10–1 × 1 MMF. (I) Photoimages exhibiting the adsorption–desorption processes. (II) Removal performance of NH_2_-MIL-101(Al)-PA12-10–1 × 1 MMF towared MB during 5 consecutive adsorption–desorption cycles. Reprinted with permission from Ref. [108]. Copyright 2019, American Chemical Society

**Fig. 13:**
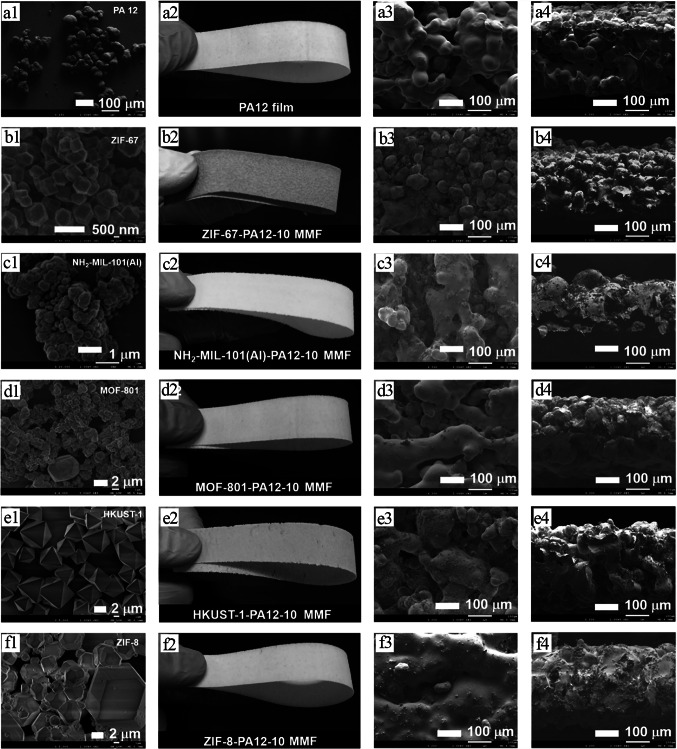
**a1–f1** SEM images of the fabricated samples. **a2–f2** photographs, **a3–f3** top surfaces, and **a4–f4** cross-sectional SEM images of the fabricated samples. Reprinted with permission from Ref. [108]. Copyright 2019, American Chemical Society

With such approach, a series of polymer-based MOF mixed matrix films (MMFs) has been prepared by Li et al. [108] utilizing thermoplastic polyamide 12 (PA12) powder as the printable polymer matrix and five kinds of powdered MOFs such as MOF-801, ZIF-67, ZIF-8, HKUST-1, and NH_2_-MIL-101(Al) as the porous fillers (Fig. 12a). Therein, single-layer MMFs with lacing structure were printed utilizing all kinds of MOFs with various MOF contents to evaluate their mechanical properties (free-standing), BET surface area, thickness, water permeability, hydrophobia, and structural stability. The SEM images of the prepared samples displayed that their sizes were in the range of ≈ 0.2–100 μm (Fig. 13a1–f1). Moreover, it was found that most of the printed MOF-PA12 MMFs can be shaped or folded, indicating their good flexibility and excellent mechanical stability (Fig. 13a2–f2). The fabricated single-layer MMFs with appropriate mechanical properties and high porosity can be applied as adsorbent materials for removing MB dye from aqueous solutions. The experimental adsorption results demonstrated that the NH_2_-MIL-101(Al)-PA12 MMF with the lacing structure and the smallest pore size can be used as an efficient adsorbent material with easy-to-collect ability, suitable adsorption kinetic, and high adsorption capacity. Moreover, the reusability of the adsorbents was evaluated by cyclic adsorption/desorption tests, in which after the adsorption process, the adsorbents were soaked in methanol for desorption of the MB molecules (Fig. 12b). As shown in Fig. 12c, after five consecutive adsorption–desorption cycles, the removal efficiencies of this adsorbent toward MB were still at acceptable value (≈ 83%), indicating its recyclability as well as durability.

### Digital Light Processing

The digital light processing (DLP) approach generally involves localized photopolymerization of monomers or oligomers with appropriate photoinitiators. On the other hand, DLP printing uses photoinduced cross-linking to solidify liquid resins containing monomer or oligomers on locally illuminated regions, replicating a 3D structure sequentially or layer-by-layer [137]. Therefore, this strategy enables rapid prototyping and provides control over the layer's thickness resolution, which is an important consideration when working with 3D-printed MOF monoliths or MOF-based mixed-matrix membranes (MOF-MMM). For instance, Pustovarenko et al. [65] demonstrated using digital light processing to prototype several MOF-MMMs rapidly. Therein, MOF-based printable ink has been formulated from post-synthetically modified MIL-53(Al)-NH_2_ with methacrylic functionality (MIL-53(Al)-NH_2_/MMA) and commercially available acrylate oligomers. Accordingly, the inks made with MIL-53(Al)-NH_2_/MMA could be rapidly converted into free-standing composite membranes with favorable shape and thickness. The superior gas separation efficiency of the fabricated 3D-printed MOF-MMMs in H_2_ and CO_2_ gas mixture with a 1:1 molar ratio demonstrated the enhanced permeability of the MIL-53(Al)-NH_2_/MMA-containing composite compared to the gas separation efficiency of the pure polymer. However, the relatively low H_2_/CO_2_ separation selectivity of these membranes compared to previously published MMMs indicated the existence of non-selective micro-voids for penetration of gas molecules surrounding the MOF nanoparticles [138]. The authors believed this strategy might be effectively used for rapid prototyping of MOF-MMMs and provide a new opportunity for future research in this area.

**Fig. 14:**
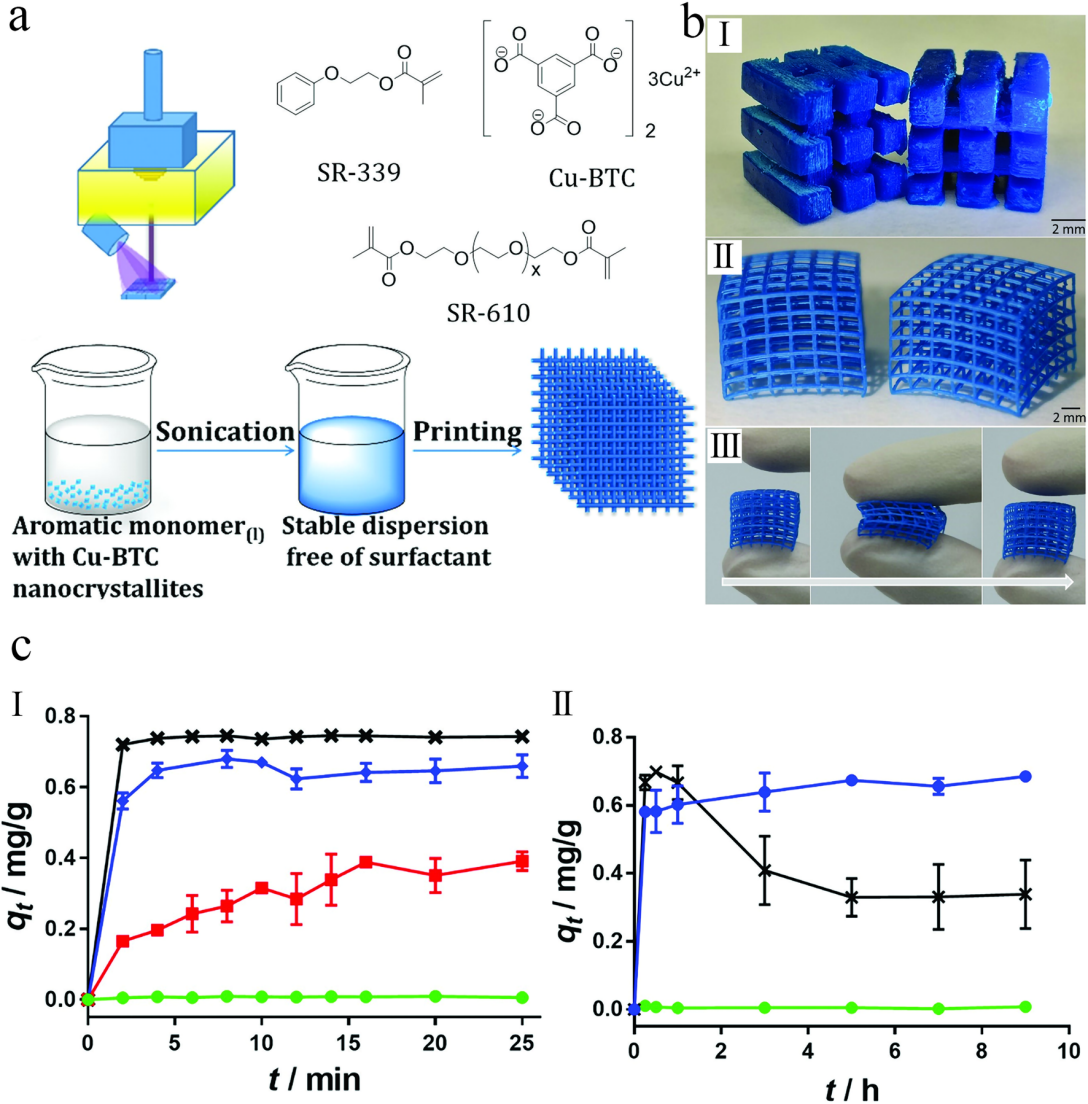
**a** Schematic illustration of the DLP strategy with the chemical structure of the used materials. **b** Photoimages of 3D-printed MOF monoliths: (I) Different 3D-printed models of HKUST-1@polymer. (II) 3D-printed nets of HKUST-1@polymer. (III) Illustration of the flexibility of the 3D-printed MOF monoliths before, during, and after pressing. **c** Dye adsorption performance of the fabricated adsorbents: (I) MB adsorption performance of HKUST-1 nanoparticles and polymer over short periods. (II) MB adsorption performance of pure HKUST-1 nanoparticles, 3D-printed HKUST-1@polymer monoliths, and polymer over long periods. Reprinted with permission from Ref. [66]. Copyright 2018, John Wiley & Sons

The DLP strategy allows the fabrication of 3D polymeric flexible monoliths incorporating MOF nanoparticles while keeping their functionality and significantly improving their hydrolytic stability. To highlight the benefits of this method, Halevi et al. [66] used the water-sensitive HKUST-1 MOF, in which its dye adsorption efficiency was carried out in water to determine the change of MOF's functionality in 3D-printed monolithic structures. Therein, the HKUST-1 nanoparticles were dispersed within a photopolymerizable composite containing 2-phenoxyethyl acrylate (Sartomer SR-339), polyethylene glycol (600) diacrylate (SR-610), and two photoinitiators (Irgacure-184 and Irgacure-819), to allow the fabrication of a composite capable of supporting the MOF nanoparticles (Fig. 14a). Accordingly, the DLP printer successfully produced the formulated ink containing HKUST-1 nanoparticles, monomers, and photoinitiators. As a result, 3D-printed MOF structures with the characteristic blue color of the HKUST-1 could be created (Fig. 14b). The addition of HKUST-1 nanoparticles with the 3D-printed MOF monoliths dramatically improved the MB adsorption capacity compared to the simple incorporation within the polymer matrix. Moreover, as shown in Fig. 14c(I), the MB adsorption capacity nearly equaled that of the pure HKUST-1 nanoparticles, with the same adsorption kinetics during the early stages of the adsorption process.

The stability and durability of the fabricated adsorbent were further studied by dye adsorption measurments over an extended period of time (≈ 9 h). As observed from Fig. 14c (II), after 30 min, the pure HKUST-1 nanoparticles adsorbed MB molecules. At later times, the adsorbed MB molecules were released back into the solution. The release of MB molecules in this experiment may be due to the structural decomposition of HKUST-1 nanoparticles via hydrolysis [139]. In contrast, the MB molecules adsorbed by the 3D-printed HKUST-1 monoliths stayed adsorbed during the whole duration, owing primarily to the increased structural stability of 3D-printed HKUST-1 in water. The authors believed that this strategy for direct 3D printing of functional monoliths incorporating MOFs represents a major step forward in developing functional devices using MOF-based materials [66].

Inspired by Halevi et al.’s work, Cherevko et al. [140] produced a composite consisting of MOF-5 through DLP method. For the composite formulation, they utilized the commercially accessible Wanhao Industrial Blend resin, and the blending of resin and MOF particles occurred at room temperature for 10–15 min using an ultrasonic homogenizer. To find the optimal composition of the composite, they gradually increased the particle concentration in the photopolymer resin, starting from 1 wt%, to determine the optimal content of MOF particles that resulted in acceptable quality of the 3D printing. At the 10 wt% of MOF-5, the manufactured 3D objects showed good spatial resolution and uniformity of 3D printing. The XRD analysis revealed that integrating MOF-5 particles into the composite during the 3D printing process did not lead to any crystalline structure loss or phase composition alterations. It was found that despite a good distribution, MOF-5 particles form some aggregated clusters whose degree depended on the time spent for homogenization. Incorporating MOF-5 particles into the photopolymer matrix reduced their ability to adsorb molecules, as evidenced by initial experiments involving the adsorption of fluorescein and eosin from aqueous solutions. Thus, the authors proposed thermal annealing of the final composite to remove a portion of the photopolymer resin and better expose MOF-5 particles to the environment.

Chaudhari and Tan [91] fabricated a white light-emitting diode (LED) by combining a dual-guest@MOF compound with a light-emitting polymer, integrating them into various 3D-printed structures using the DLP technique. They blended the "A + B@ZIF-8" powder, where A represents fluorescein and B signifies rhodamine B, with a blue-emitting photopolymer resin to form a composite material. This composite was subsequently employed to fabricate diverse objects via digital light processing. These objects were designed to emit white light when subjected to UV irradiation. Among the demonstrated creations were disc-shaped pellets, which emitted warm white light upon exposure to a 400 nm UV LED. Furthermore, they demonstrated the ability to adjust the chromaticity of the emitted light by methodically altering the thickness of the 3D-printed pellets. This enabled the creation of various color temperatures ranging from cool to warm white light. The authors suggested that the light emission capability arises from the structural relaxation of the "A + B@ZIF-8" compound when it disperses within the photopolymer resin. The capability to adjust the emitted light's color by modifying the thickness of the 3D-printed pellets paves the way for designing photonic sensors, optoelectronics, and forthcoming metamaterials. This study also showcases the prospect of improving the durability and light resistance of functional devices by producing 3D-printed composite objects.

## Water Treatment Applications of 3D-Printed MOF Monoliths

Water contamination due to growing urbanization has become a major global issue in the last century [141, 142]. Moreover, the need for potable water has increased significantly as the impacts of global warming have become a serious concern to civilization. Emerging water pollutants such as organic dyes, heavy metal ions, persistent organic pollutants (POPs), pesticides, fluoride, pharmaceuticals and personal care products (PPCPs), phosphate, endocrine-disrupting chemicals (EDCs), etc. are typically found in secondary effluent from wastewater treatment plants and more importantly in natural water sources [10, 141, 143, 144]. Therefore, water treatment systems have become increasingly crucial for recovering and recycling available unusable water sources. Environmental nanotechnology can help meet the requirement for clean, drinkable water, because of the exceptional adsorption, catalytic/photocatalytic, and detection properties of functionalized materials [53, 145].

### Heavy Metal Ions Detection

Heavy metal ions are regarded as one of the most significant cancer-causing substances and non-biodegradable contaminants due to their high toxicity, stability, and tendency to accumulate in human organs, mostly via the food chains [146]. Therefore, for environmental and health protection, developing sensitive and routine techniques appropriate for on-site monitoring of hazardous heavy metal ions and other water pollutants [147]. Various spectrometric methods are used to measure the concentration of heavy metal ions in water. Still, they need bulky and expensive instrumentations and require expert and well-trained technicians, which limits their on-site applications [148]. Anodic stripping voltammetry (ASV), with its simple operation protocols, reasonably high sensitivity, and low price, is viable for heavy metal ion identification. However, conventional ASV, particularly because of modifying electrodes and designing devices, suffers from the long-lasting pre-electrolysis procedure, huge consumption of sample solutions, and poor reproducibility [58].

Nowadays, materials that have dual functionality of adsorption and sensing of heavy metal ions are quite appealing, and MOFs have lately been widely investigated in this area. However, the application of MOFs as electrode materials/modifiers for heavy metal detection can be considered undiscovered since just a few studies addressing the voltametric identification of heavy metal ions have been reported in the published papers, mainly due to their poor water stability and electronic conductivity [130, 149]. MOF-based electrodes are typically made in a multi-step process that begins with the drop-casting of MOFs onto the surface of a glassy carbon electrode (GCE) [149]. Before each test is performed, the surface of each bare GCE must be polished and washed in various solutions via sonication. Eventually, the MOFs are dripped onto the GCE and let to dry naturally. Thus, these MOF electrodes made via drop-casting cannot be considered ready-to-use or stand-alone devices.

**Fig. 15:**
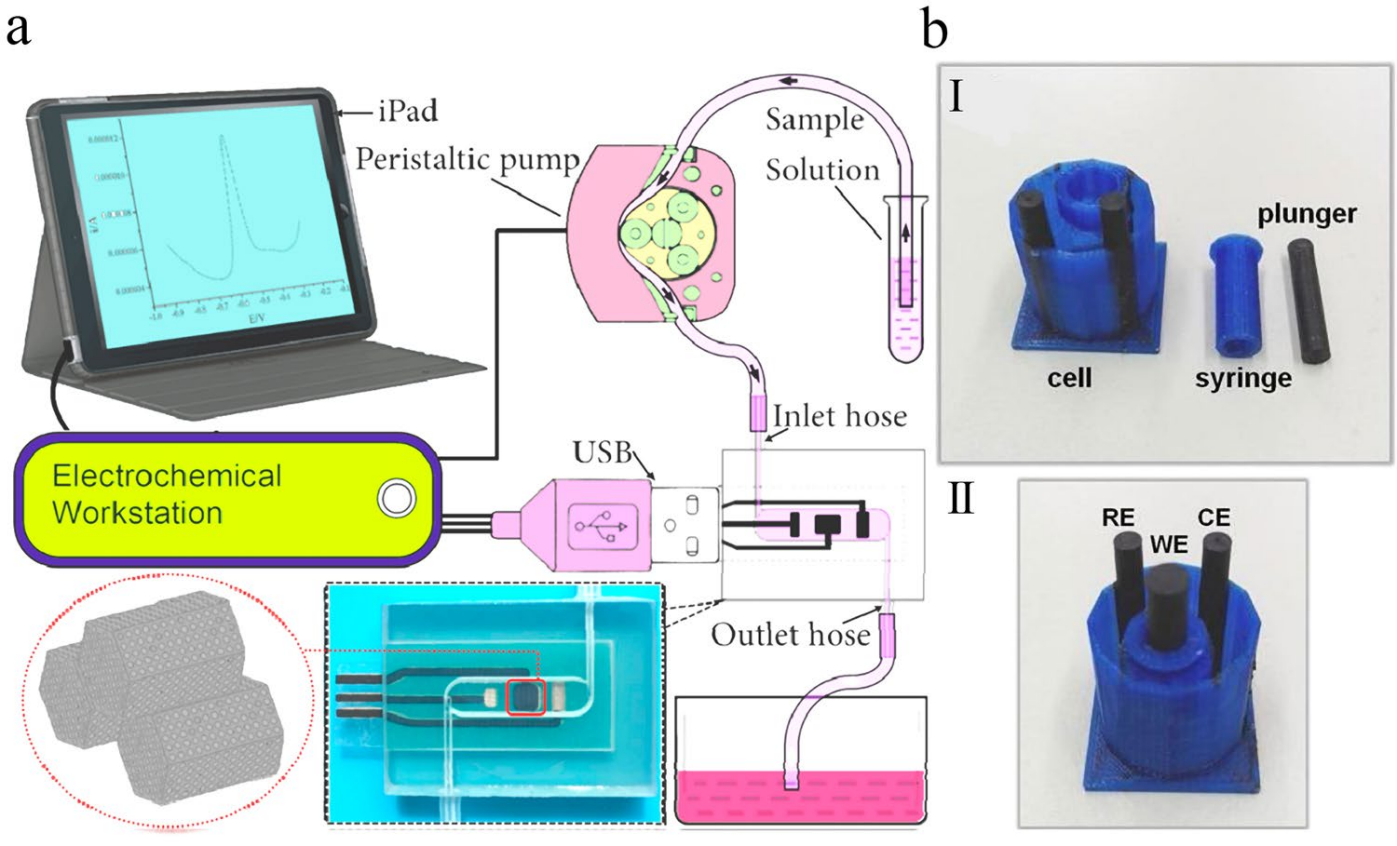
**a** Schematic illustration of the detecting system for heavy metal ions. Reprinted with permission from Ref. [58]. Copyright 2016, American Chemical Society. **b** Photoimage of (I) the main parts of the 3D-printed monolith and (II) the complete 3D-printed monolith. Reprinted with permission from Ref. [85]. Copyright 2020, Elsevier

To address the abovementioned challenges, 3D printing was adopted as a fabrication tool to prepare a novel detecting system. 3D printing is an environmentally benign and cost-effective technology that offers tremendous potential for device standardization and mass manufacturing. Based on this strategy, Hong et al. [58] employed a 3D printing technique to create a microfluidic electrochemical sensor for real-time determination of heavy metals (e.g., Cd^2+^ and Pb^2+^) from water (Fig. 15a). Therein, Mn-based MOF (Mn-MOF) was effectively synthesized and used as a precursor for the fabrication of porous Mn_2_O_3_, which significantly enhanced the active electrochemical surface and the real-time stripping detecting characteristic of heavy metal ions. In conclusion, the manufactured system had a detection limit of 0.2 µg L^−1^ for Pb^2+^ and 0.5 µg L^−1^ for Cd^2+^, which was roughly 50 and 6 times smaller than the World Health Organization's (WHO) recommendation limits [150].

Moreover, it was reported that the procedure of combining a tiny quantity of MOF particles with graphite paste (GP) to form a working electrode (WE) is much easier than the drop-casting strategy because the surface of the fabricated WE is regenerated using a syringe plunger sliding pressure, leading to the stand-alone sensors [85, 151]. Accordingly, Kokkinos and coworkers created a new integrated lab-in-a-syringe system for the quantitative designation of Pb^2+^ [151] and Hg^2+^ [85] in bottled water and spiked fish oil samples utilizing an extremely effective MOF (Ca-MOF) as an electrode modifier and 3D printing method (Fig. 15b). The lab-in-a-syringe gadget is made up of a tiny cell printed from a non-conductive PLA filament (PLA/F) and two electrodes printed on the edges of the vessel from a conductive carbon-based PLA/F, such as counter (CE) and pseudo-reference (RE). This system also includes a tiny detectable 3D-printed syringe made of non-conductive PLA/F and modified with Ca-MOF/GP, which acts as the WE. The conductive plunger produces the electrical contact between the WE and the potentiostat, which is produced from conductive PLA/F. The high adsorption performance of the Ca-MOF toward Hg^2+^ ions enhanced the sensitivity of the fabricated electrode for determination of Hg^2+^ ions with a small detection threshold of 0.6 µg L^−1^, which was similar to or less than other sensors (e.g., gold, plastic 3D-printed, and MOF-based electrodes) [149]. Additionally, this sensor exhibited a small detection threshold of 0.26 µg L^−1^ for Pb^2+^ ions. The authors believed that the proposed sensor is a promising sensor for on-site monitoring of heavy metal ions mainly due to its desirable chemical activity, the facile renewal of the electrode surface, and the quick and low-cost 3D-printed production technique.

### Oil/Water Separation

**Fig. 16:**
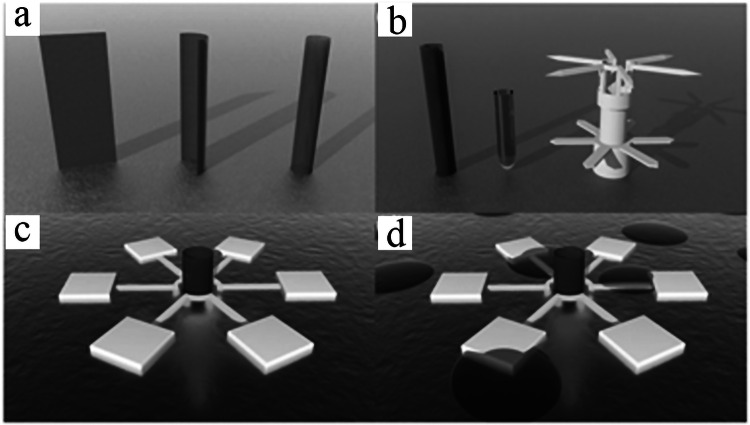
3D-printed superhydrophobic/oleophilic oil recycling system schematic. **a** Bending of the membrane to tube form, **b** rolled membrane, recycling tube, and thermoplastic printed floating device, **c** self-floating oil recycling device, and **d** oil recycling process of the device. Reprinted with permission from Ref. [154]. Copyright 2019, John Wiley & Sons

Pollution from oil has a high cost to the environment worldwide because a large amount of oily wastewater is produced by commercial, ever-increasing industrial, domestic discharge, and frequent oil spill accidents [152]. Therefore, developing environmentally friendly, energy-efficient, and sustainable oil/water separation methods is critical for removing the oil from the environment [104, 153]. In past years, super-hydrophobic and subaqueous superoleophobic membranes have received a lot of interest for their efficacy in treating oily wastewater. Compared to the adsorption processes, the membrane separation technique usually provides better reusability for the recycled oil and membranes [154]. Although several strategies have been applied to fabricate such membranes, 3D printing is the best solution to overcome the drawbacks of conventional manufacturing methods [155]. Li et al. [154] introduced an innovative oil recycling approach that recycles oil based on weight via an oil-selective membrane. As a result, without the need for any solvents or wet procedures, functional superhydrophobic graphene can be directly printed as an additive on the surface of porous nickel foams. As observed in Fig. 16, the superhydrophobic/oleophilic membrane could stay afloat on the wastewater surface. Thus, the nearby oil may spontaneously diffuse into the membrane due to gravity, whereas water molecules are unable to penetrate due to the superhydrophobic confinement's surface tension.

**Fig. 17:**
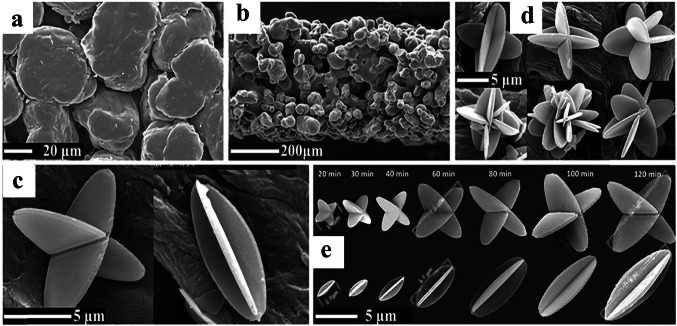
SEM images of the fabricated materials. **a** Bottom and **b** cross-sectional SEM images of the 3D PA membrane. **c** Leaf-crossed ZIF-L, **d** flower-like ZIF-L, and **e** synthesis of leaf-crossed ZIF-Ls with different reaction times. Reprinted with permission from Ref. [156]. Copyright 2019, Royal Society of Chemistry

Yuan et al. [156] used a similar technique to create a hierarchically micro/nanoscale structured surface with superhydrophobic and underwater superoleophobic properties using an easy two-stage design of a novel 3D multi-scale ZIF-L on a 3D-printed membrane. This strategy entails synthesizing two ZIF-Ls, one of which was created by employing an aqueous solution containing a high amount of 2-methylimidazole linker (Hmim) and Zn^2+^ ions, resulting in a 3D leaf-crossed MOF. While the second one was synthesized by growing tiny needle-shaped and rod-like ZIF-Ls on the top of leaf-crossed ZIF-L (Fig. 17). An ideal multi-scale micro/nanostructural membrane is produced due to a two-step depositing of that multi-scale ZIF-Ls on a coarse 3D-printed PA membrane. The manufactured membranes displayed exceptional superhydrophobicity after being coated with polydimethylsiloxane (PDMS), with a small water contact angle of 1.56° and a static water contact angle of 158.6°. Both fabricated membranes showed an excellent separation performance toward oil/water separation, with over 99% oil rejection and over 24,000 L (m^−2^ h^−1^) oil flux. The authors expected that this work would provide a novel way for fabricating a superhydrophobic membrane for oil/water separation using 3D printing and the creation of micro/nanostructural ZIF-Ls.

### Organic Dye Removal Via Adsorption Processes

**Fig. 18:**
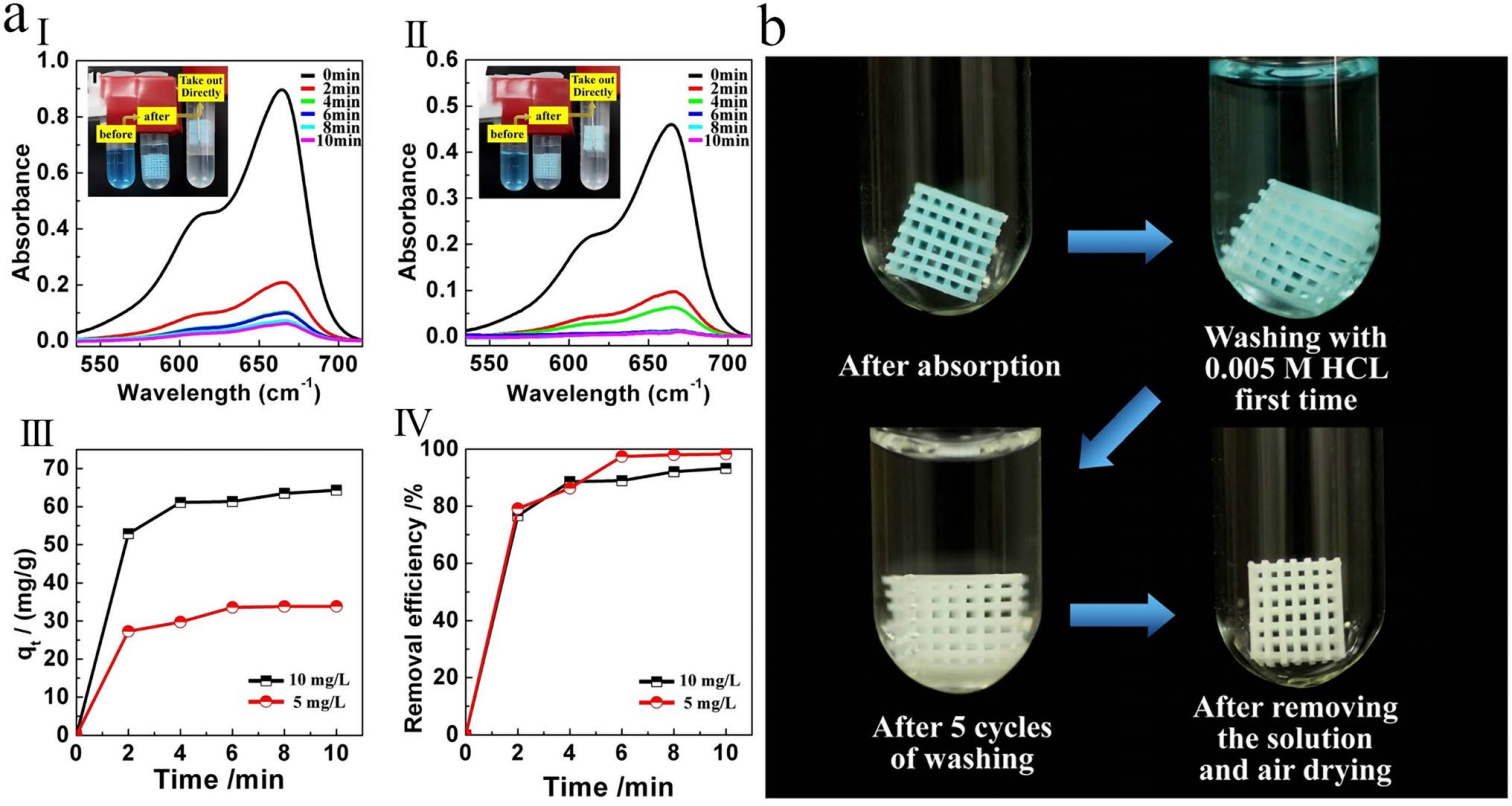
**a** Adsorption performance results: UV–Vis spectra of (I) 10 and (II) 5 ppm MB solutions during the adsorption process using the Cu-BTC/ABS-8 composite (insets show the photographs of 10 and 5 ppm MB solution before and after adsorption process). (III) MB adsorption capacities as a function of time. (IV) MB removal efficiency as a function of time. **b** Photoimages of the regeneration process of Cu-BTC/ABS composite during MB removal. Reprinted with permission from Ref. [54]. Copyright 2014, Nature Publishing Group

The 3D-printed MOF-based nanocomposites have also showed exceptional adsorption performance toward water pollutants. For instance, Wang et al. [54] modified an ABS framework with a coating of Cu-BTC MOF (BTC = benzene tricarboxylic acid) porous structure via an efficient step-by-step in situ synthesis method. A 3D printer was used to make MOF-coated polymer composites more flexible. The fabricated Cu-BTC/ABS composite was applied as an adsorbent material to remove MB dye from water. The Cu-BTC/ABS composite could occupy most of the tubular reactor's available area, allowing for successful MB adsorption without any need for stirring. As observed from Fig. 18a, MB was eliminated from water solutions in a short period, which its removal efficiencies reached 93.3% and 98.3% for the solutions containing 10 and 5 ppm MB, respectively, only after 10 min. Accordingly, Cu-BTC/ABS-8 had maximal adsorption capabilities of 64.3 and 33.9 mg g^−1^ for 10 and 5 ppm of MB dye solutions, respectively (Fig. 18a(III)). This composite also has the advantage of being easily regenerated by washing with diluted HCl solution and being used for more than five cycles with a 65%–95% removal efficiency of MB (Fig. 18b).

**Fig. 19:**
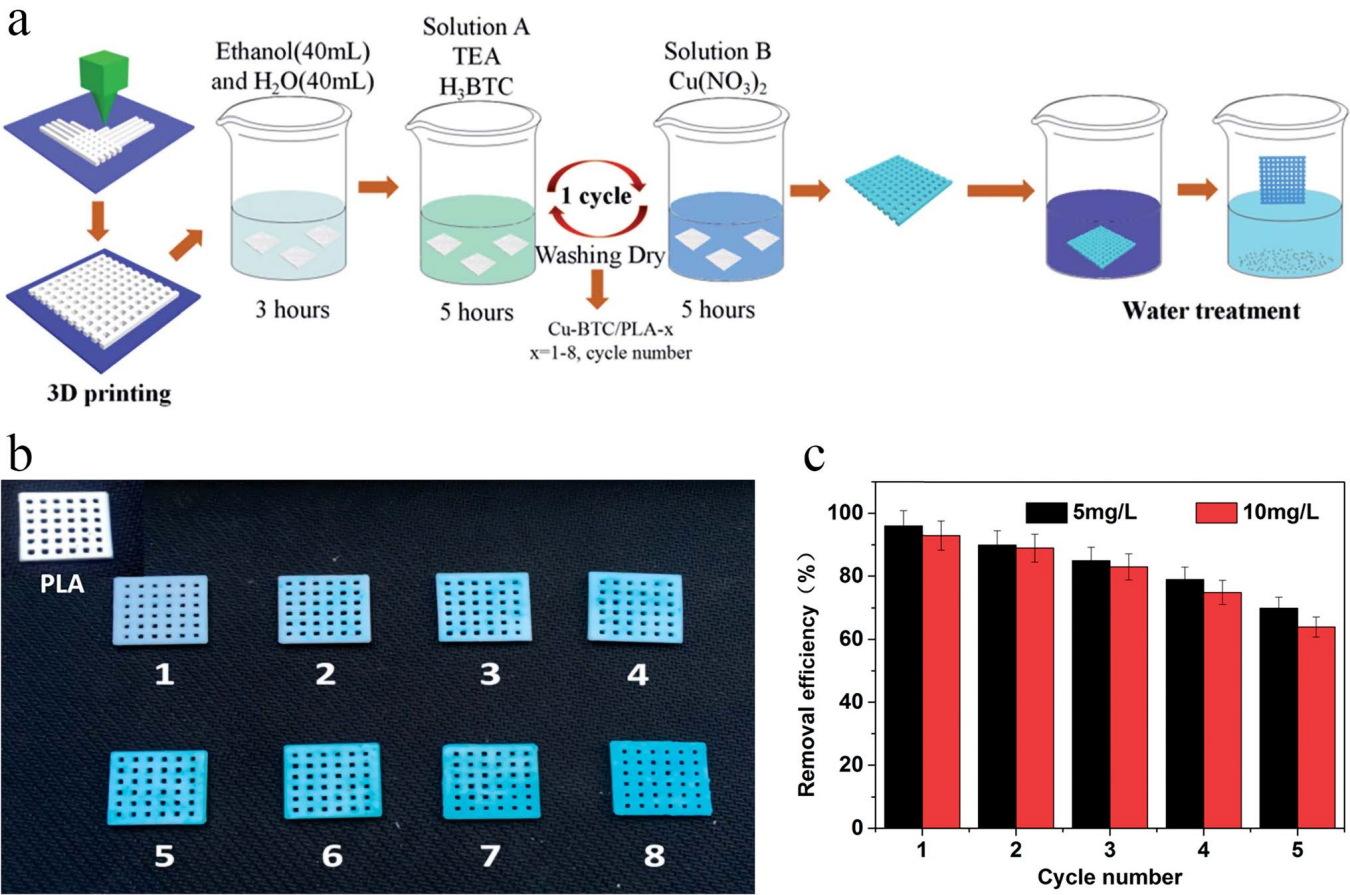
**a** Schematic representation for the preparation processes of Cu-MOFs/PLA composite. **b** Photo images of the fabricated Cu-MOFs/PLA-x (x = 1–8) composites with various layers of the Cu-MOFs particles. **c** Removal performance of the regenerated Cu-MOFs/PLA composites after regenerating with acetone treatment. Reprinted with permission from Ref. [157]. Copyright 2017, Royal Society of Chemistry

In another study, Shi et al. [157] established a new technique for the fabrication of Cu-MOFs/PLA film to remove MG dye from wastewater. Therein, a simple method of fabricating the Cu-MOFs/PLA composites was used, including the in situ gradual growth of porous Cu-based MOF on the external surface of a 3D-printed PLA structure (Fig. 19). This strategy not only makes the Cu-MOFs/PLA composites more stable but also makes it easier to reuse and recycle them. Instead of separating and regenerating MOF-based adsorbent materials, this strategy avoids these time-consuming steps. The removal performance of the manufactured Cu-MOFs/PLA composites toward MG adsorption was thoroughly studied, with a high efficiency of > 90% after just 10 min. Furthermore, following a simple acetone wash, the Cu-MOFs/PLA composites were shown to be reusable more than five times. Accordingly, as observed in Fig. 19c, the removal efficiency of Cu-MOFs/PLA composites for MG was maintained at over 80% after three consecutive adsorption–desorption cycles. Even after five cycles, this parameter was found to be more than 60%. Because of the chemical adsorption of dye molecules onto MOFs, the removal efficiency of composites toward MG may be reduced. This chemical adsorption is difficult to remove by acetone washing.

**Fig. 20:**
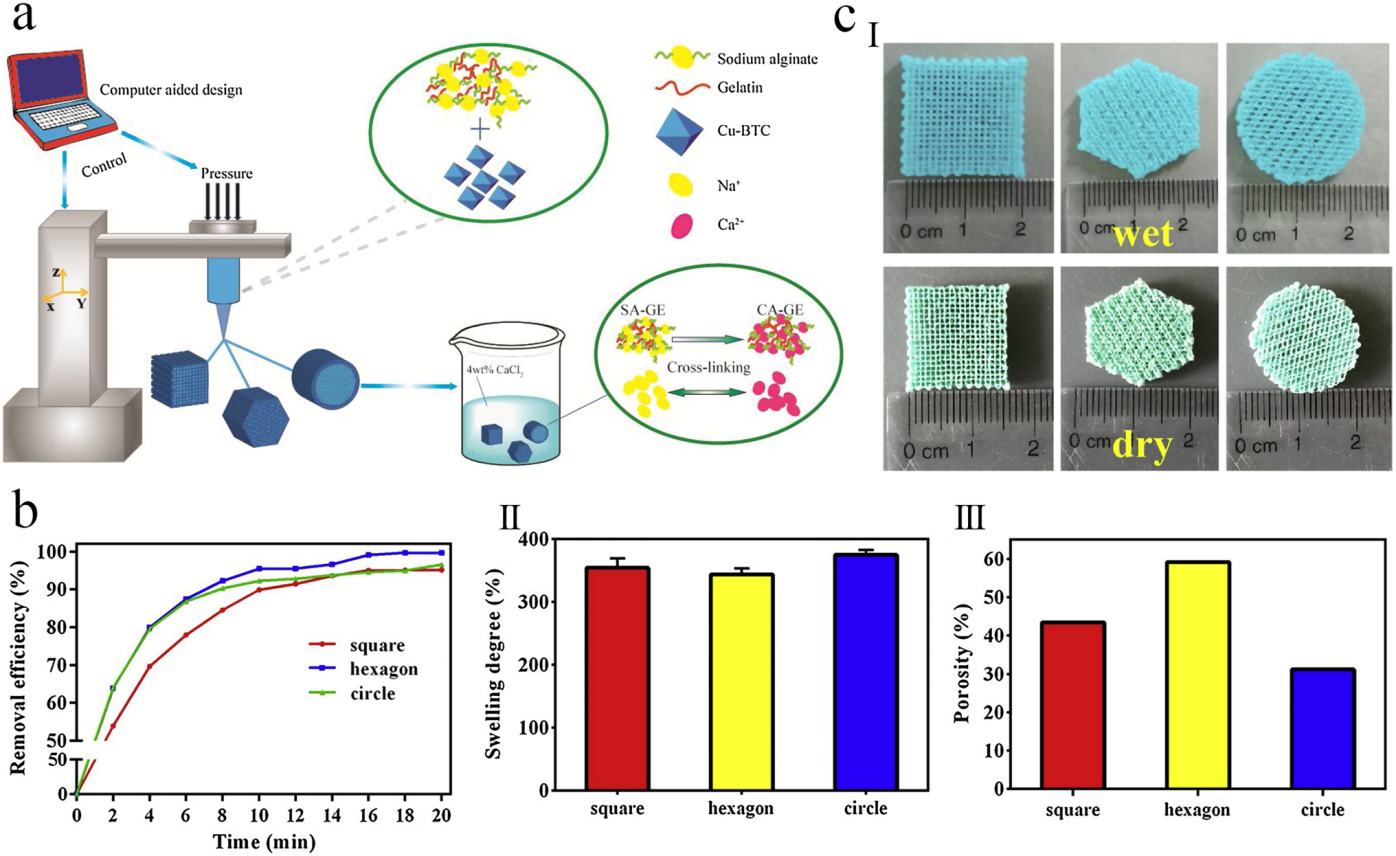
**a** Fabrication processes of 3D-printed MOF/CA-GE samples with three different structures. **b** MB adsorption performances of fabricated samples with different structures as a function of time. **c** Characterizations of the 3D-printed MOF/CA-GE samples with various structures: (I) the photoimages of three printed structures in a wet (top) and dry (bottom) state, (II) the equilibrium swelling degree of samples with three different printed structures, and (III) the porosity of different samples with three printed structures. Reprinted with permission from Ref. [94]. Copyright 2020, Elsevier

Usually, 3D-printed MOFs can be molded into various shapes using the DIW approach, which avoids the high-pressure drop and instability of MOF powders in water treatment applications. Zhan and coworkers [94] fabricated 3D-printed skeleton adsorbents based on calcium alginate/gelatin as a biocompatible binder and Cu-BTC MOF as a porous filler via DIW strategy. Therein, the Cu-BTC MOF with different morphologies and sizes was firstly synthesized, and then the resulting MOFs were combined with gelatin and sodium alginate (the SA-GE matrix) to produce a printable ink for the subsequent 3D printing process. Consequently, three designs were created: circle, hexagon, and square, and the printed skeletons were instantly cross-linked with CaCl_2_ solution for mechanical stability (Fig. 20a). Finally, the effects of the Cu-BTC loadings, the printing geometry, and the size/morphologies of Cu-based MOF on the adsorption efficiency of MOF/calcium alginate and gelatin (CA-GE) samples toward different dyes (e.g., MB, MG, methyl violet (MV), auramine O (AO), and rhodamine B (RB)) were systematically investigated. As a result, it was proposed that the Cu-BTC intrinsic microporous structure and the 3D matrix's meso/macroporous morphologies led to the high adsorption efficiency of MOF/CA-GE samples toward the aforementioned organic dyes and their mixtures. Compared to other existing solids, it was discovered that the square solid showed a comparatively sluggish adsorption efficiency (Fig. 20b). While the hexagonal sample exhibited the best adsorption efficiency towards these organic dyes, mainly due to its highest porosity and smallest swelling ratio (Fig. 20c) [158]. More importantly, the fabricated MOF/CA-GE monoliths could be recovered by soaking them in HCl solution for 60 min, allowing them to be utilized for at least seven times after that regeneration.

Radionuclides are used extensively in hospitals for medical imaging and radiotherapy. Among them, radioiodine (^131^I) is widely utilized in nuclear medicine, particularly for treating thyroid cancer and hyperthyroidism [159]. Therefore, following its medical use, radioiodine-containing wastewater is usually held in storage vessels till its radioactivity decreases to acceptable levels. However, the frequent usage of this material in hospitals increases the expense and time required for waste handling. Moreover, a considerable quantity of medically produced ^131^I has been discovered in wastewater, necessitating the development of adequate analytical methods for its detection and treatment [160].

As a representative work, del Rio et al. [161] synthesized a porous silver-functionalized UiO-66 (UiO-66-So_3_H@Ag) framework with uniform Ag distribution via a facile one-pot solvothermal method. The fabricated functionalized MOF exhibited exceptional extraction capacity (≈ 1 MBq g^−1^) toward ^131^I, which was much higher than that of the pristine UiO-66-SO_3_H framework, owing mostly to the strong affinity of Ag particles for iodide. Furthermore, the synthesized MOF particles were combined into a 3D-printed structure for actual extraction procedures, employing polyvinylidene fluoride (PVDF) as a matrix. The resulting 3D UiO-66-So_3_H@Ag/PVDF monolith with exceptional performance and reusability extracted ^131^I from hospital waste and polluted water samples with recoveries over 90% in all tests, indicating that it possesses unique properties for treating actual wastewater samples.

**Fig. 21:**
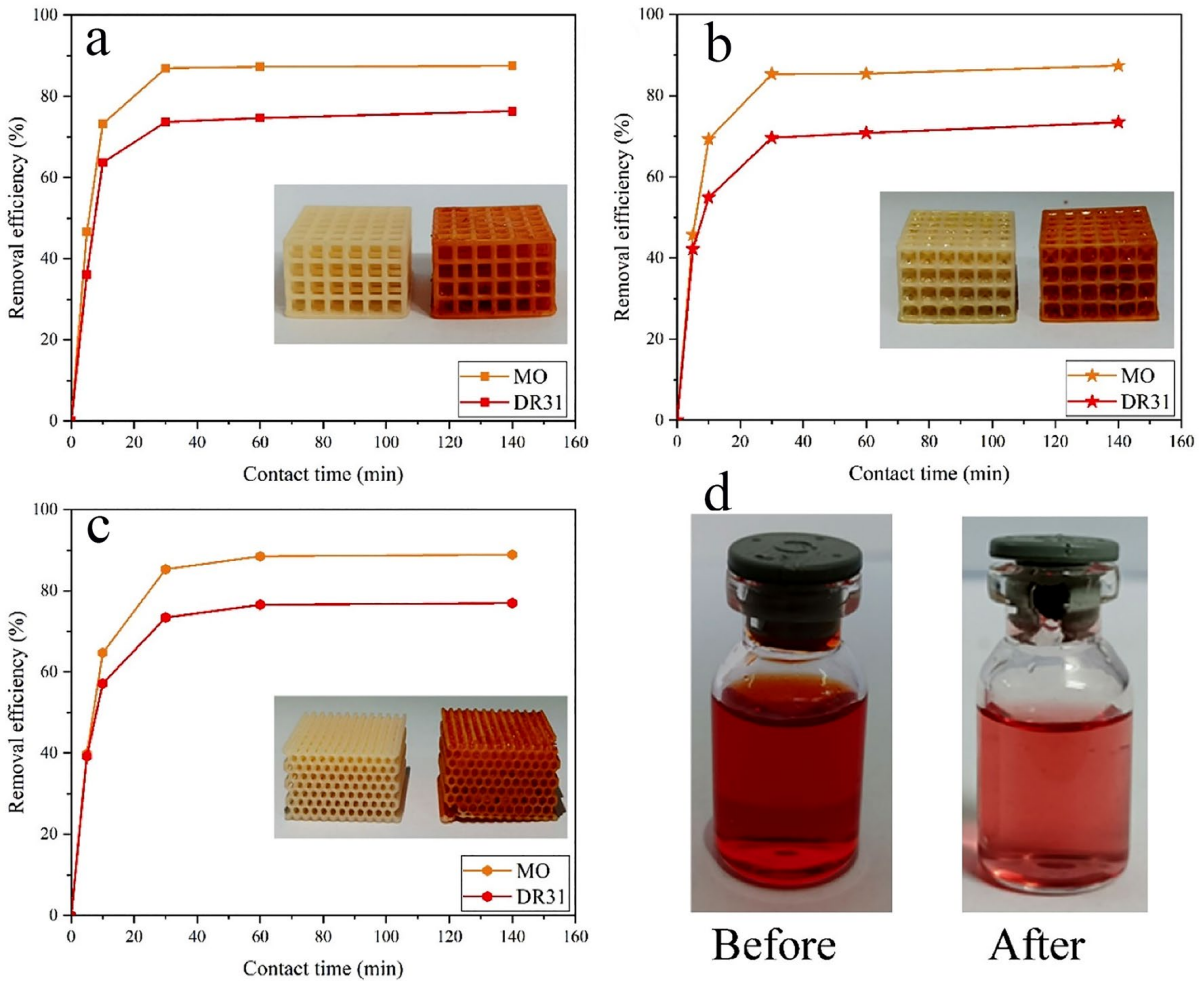
Removal efficiencies of 3D-printed MXene/UiO-66 monoliths with different geometries of **a** grid, **b** star, and **c** honeycomb toward MO and DR31. **d** Digital images of dye solution before and after adsorption. Reprinted with permission from Ref. [162]. Copyright 2023, Taylor & Francis

More recently, Shahriyari Far et al. [162] prepared a series of 3D-printed MXene/UiO-66 monoliths with various shapes of honeycomb, star, and grid through DLP strategy, then applied them as adsorbent to eliminate different organic dyes from water efficiently. Due to the excellent structural stability and hydrophilic surface of MXene nanosheets, along with the high porosity and large surface area (1215 m^2^ g^−1^) of UiO-66 MOFs, the resulting 3D-printed MXene/UiO-66 monoliths exhibited good adsorption performances toward both cationic and anionic dyes. Accordingly, the 3D-printed MXene/UiO-66 monolith with honeycomb geometry showed the highest removal efficiencies of 88.95% and 76.98% toward MO and direct red 31 (DR31), respectively (Fig. 21). Moreover, these monoliths exhibited easily recoverable properties, good reusability, and excellent stability after four consecutive adsorption–desorption cycles, indicating their capability for long-term water treatment applications.

### Pollutant Degradation Via Catalytic/Photocatalytic Processes

Recently, MOF-based materials have received great attention for water purification. However, costly and time-consuming separation processes are one of the main important challenges of MOF-based catalysts/photocatalysts employed for wastewater purification [163]. Therefore, the fabrication of hybrid materials based on MOF photocatalysts and supporting materials can overcome the abovementioned challenges [61]. In particular, immobilizing heterogeneous MOF catalyst/photocatalyst materials on porous supporting materials can significantly improve their separation after reactions [102]. It was reported that using polymers as substrates for the immobilization of MOF crystals offers numerous advantages. Usually, polymers are lightweight, resistant to oxidation, chemically inert, largely UV transparent, and inexpensive. Additionally, the recent progress in 3D printing makes it possible to form various polymeric supports with controlled and adjustable topographies and morphologies. For instance, 3D printing technology is used to fabricate fractal materials, which is a novel and emerging technique in polymeric substrate formation [164]. This technique aims to create a substrate with a chemically active surface area, which can attach more catalyst crystals on its external surface and increase the interfacial interaction between catalyst crystals and substrate [165].

Based on this strategy, Li et al. [166] demonstrated a novel method that enables to immobilization 100% of the catalyst crystals onto the external surface of the polymeric substrate. A series of 3D-printed hybrid photocatalysts were prepared through plasma grafting of TiO_2_, ZnO, and Fe-BTC MOF crystals onto a fractal-inspired 3D-printed substrate. The photocatalytic performance of the fabricated hybrid photocatalysts was investigated through the photodegradation of ciprofloxacin antibiotic for Fe-BTC MOF, and RB dye for ZnO and TiO_2_ under simulated sunlight irradiation. The fabricated PLA/Fe-BTC MOF hybrid showed good removal efficiency (≈ 75%) toward ciprofloxacin antibiotic, associated with its adsorption ability and photocatalytic degradation performance. It was found that the Fe-BTC MOF remains active after being covalently attached to the PLA substrate via the plasma-induced grafting method, confirming that both pre-functionalization and the plasma grafting stages do not change the functionality as well as the crystalline and porous structures of MOFs. The authors believed that the plasma grafting process could be employed for other MOFs.

**Fig. 22:**
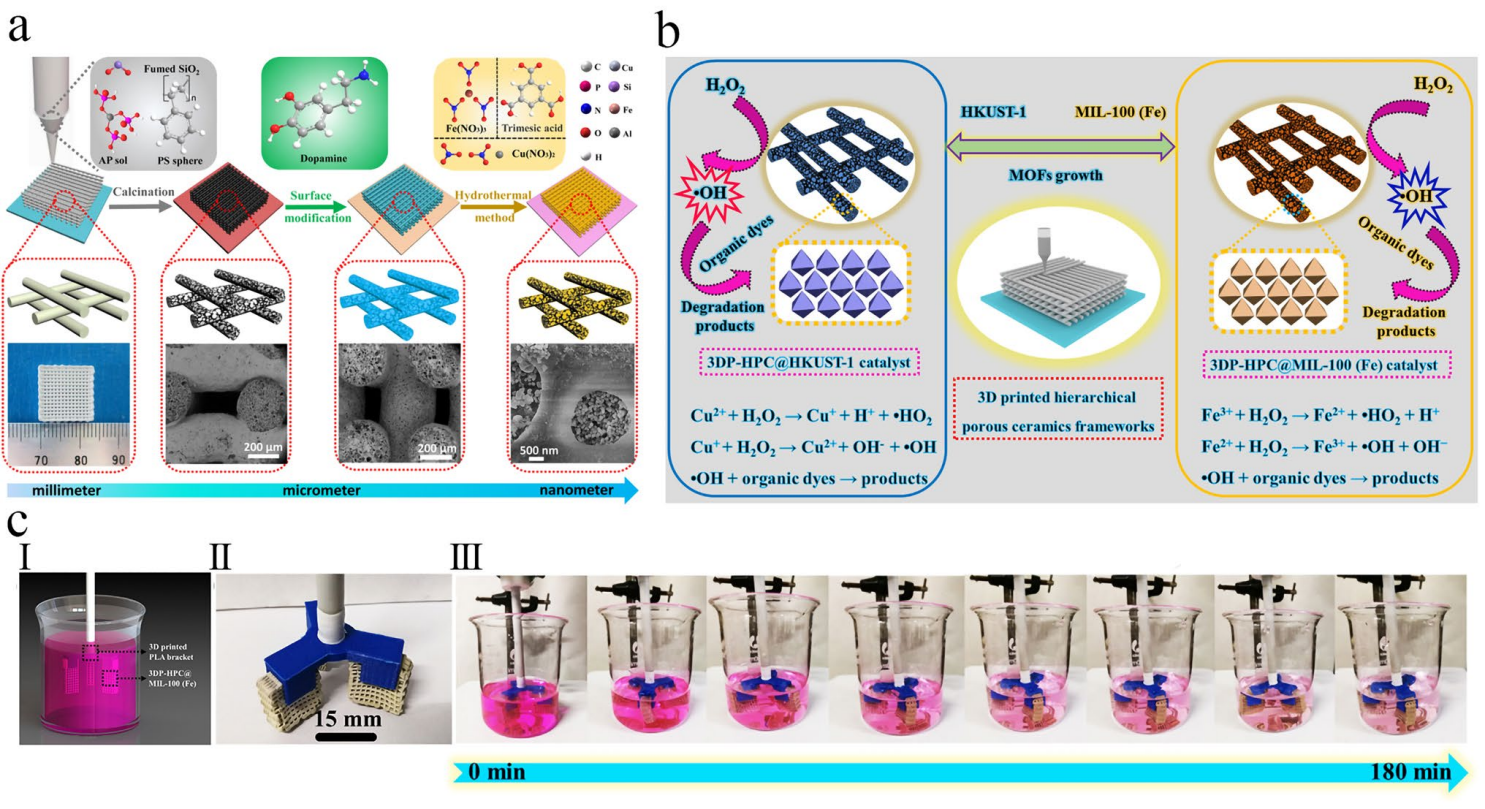
**a** Chematic illustration for the fabrication of hierarchical porous ceramics by DIW and in situ MOF growth methods. **b** Proposed catalytic degradation mechanisms for the removal of various organic dyes by the fabricated catalysts. **c** Model of sewage treatment with 3D-printed impeller agitator: (I) Schematic of 3DP-HPC@MOFs impeller agitator for the catalytic degradation of RB dye. (II) All-3D-printed impeller agitator with DIW printed 3DP-HPC@MIL-100(Fe) and fused deposition modeling printed top fixture. (III) Photoimages of the RB solution contain the fabricated catalyst as a function of time. Reprinted with permission from Ref. [167]. Copyright 2020, Elsevier

In addition, 3D-printed porous ceramics would be the other promising substrate for immobilizing MOF crystals. For example, a pioneering work garnished 3D-printed porous ceramics with different MOF nanoparticles by a facile hydrothermal process (Fig. 22a) [167]. It demonstrated a versatile approach for fabricating chemically reactive 3D-printed hierarchical porous ceramics (3DP-HPC) garnished with different MOF particles. In this respect, hierarchical porous ceramic monoliths with adjustable porosity were created using a fumed silica (SiO_2_)-based ink with superior rheological and thixotropic characteristics. Various MOFs, such as HKUST-1 and MIL-100(Fe), were loaded by in situ method onto the framework of 3D-printed ceramics to increase their catalytic activity. The fabricated 3D-printed catalysts with hierarchical features exhibited excellent performance for degrading several organic dyes (e.g., MB, MG, RB, and CV) based on the Fenton reaction, mainly due to their high surface area, hierarchical channels, and abundant active sites in spongy systems. Additionally, the fabricated 3DP-HPC@MOFs catalysts displayed superior cyclical stability and manipulating properties, allowing them to be directly separated from treated water without requiring a lengthy and complicated separation process, demonstrating their suitability for sustainable wastewater treatment applications. Accordingly, the 3DP-HPC@HKUST-1 catalyst showed the highest degradation rate and removal efficiency of 0.2709 min^−1^ and 99.68%, respectively, toward MG dye. More importantly, the dynamic catalytic device showed higher removal efficiency toward organic dyes.

Based on the proposed degradation mechanism (Fig. 22b), the ·OH radicals are the main highly reactive species that degrade hazardous organic dyes into smaller molecules. Additionally, they fabricated another 3D-printed continuous dynamic catalytic reactor-agitating impeller (Fig. 22c), in which the impeller skeleton was fabricated by FDM strategy and merged with three large-scale 3DP-HPC@MOFs supported catalysts. Accordingly, as shown in Fig. 22c(III), this device was able to degrade RB efficiently over time, and the contaminated water almost became colorless after 3 h.

## Gas Adsorption/Separation Applications of 3D-Printed MOF Monoliths

Compared to other traditionally shaped adsorbent materials like pellets or beads, monolith frameworks result in less pressure loss during adsorption processes and better heat and mass transmission [133]. Accordingly, these materials allow higher gas velocities, leading to substantially shorter adsorption–desorption cycle durations. Therefore, monolithic structures are becoming increasingly essential in gas adsorption due to the necessity for short cycles [168]. More importantly, monolithic contactors exhibited great potential for practical gas adsorption applications, mainly due to the exceptional geometry and parallel channels, resulting in high gas throughput, uniform flow pattern, attrition-free systems, and low-pressure drop [169]. As a result, many articles on 3D-printed adsorbent materials for gas storage have been written in the previous decade, demonstrating the increased interest in this area of study [88, 170–172].

### CO_2_ Capture

Carbon dioxide (CO_2_) is the most common greenhouse gas produced by human activity, with an average concentration of around 400 ppm [173]. Moreover, even if CO_2_ emissions remain unchanged for the next three decades, the atmospheric concentration of CO_2_ is predicted to reach 550 ppm by 2050. Generally, the increase in the atmospheric concentration of CO_2_ may increase global average temperature, changes in snow and ice cover, and a reduction in the upper ocean pH. Therefore, the capture and storage of this greenhouse gas from its primary emission sources are very important in controlling its atmospheric concentration [174, 175].

MOFs have also gotten much attention in gas adsorption, particularly CO_2_ capture, because of their huge surface area, high porosity, good chemical and thermal stability, low density, and high adsorption capacity [176]. However, for practical gas adsorption applications, powdered MOFs must be converted into a robust structure, including foams, beads, monoliths, pellets, etc. [177]. For instance, Hong et al. [178] demonstrated the fabrication of MOF-based monoliths and investigated their gas adsorption performance, particularly emphasizing biogas upgrading applications. MIL-101(Cr) was chosen as porous adsorbent due to its stability in ambient situatian, liquid water, and different chemical solvents [179]. Moreover, this MOF exhibited an excellent CO_2_ adsorption capability, ranging from 22.9 mmol g^−1^ at room temperature and 30 bar to 40 mmol g^−1^ at room temperature and 500 bar [180]. The experimental adsorption isotherms demonstrated that the CO_2_ adsorption capacity for purified MIL-101(Cr) monolith (0.91 mmol g^−1^) was significantly lower than that of the purified MIL-101(Cr) nanoparticles (1.44 mmol g^−1^), mainly due to the existence of bentonite in the monolith's structure which acts as a binding agent. More interestingly, the adsorption flow breakthrough studies revealed that this MOF monoliths could be entirely regenerated at 423 K for several adsorption runs with no change of adsorption effiiency [178].

**Fig. 23:**
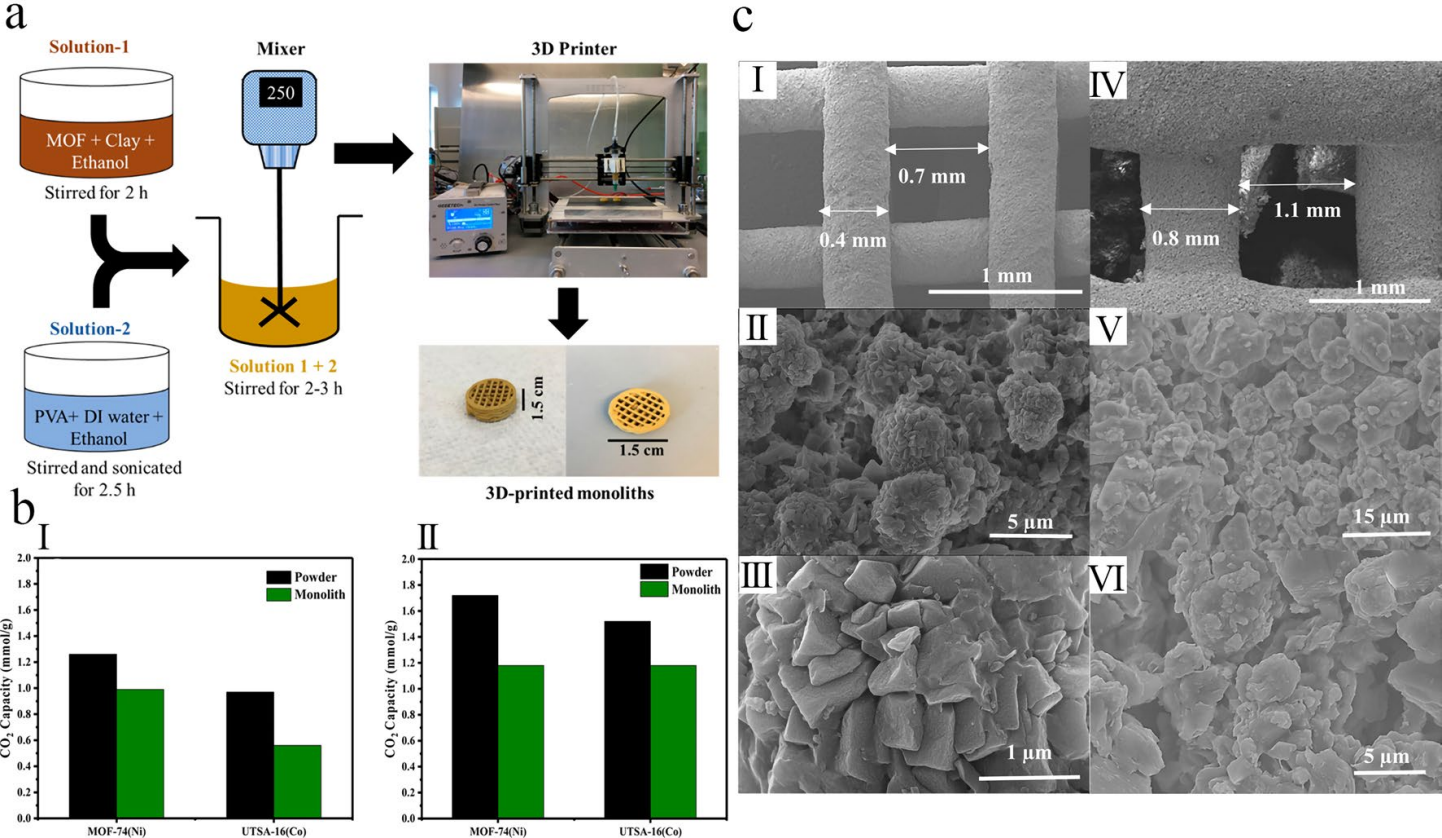
**a** Schematic illustration of the manufacturing procedure of 3D-printed UTSA-16(Co) and MOF-74(Ni) monoliths. **b** CO_2_ adsorption capacities of the fabricated 3D-printed MOF monoliths and their corresponding MOF nanoparticles under (I) 3000 and (II) 5000 ppm CO_2_/N_2_ at room temperature and 1 bar. **c** SEM images of the 3D-printed (I-III) MOF-74(Ni) and (IV–VII) UTSA-16(Co) monoliths. Reprinted with permission from Ref. [55]. Copyright 2017, American Chemical Society

Rezaei’s group, as a pioneer research group in this field, fabricated various kinds of 3D-printed MOF-based monoliths and then examined their CO_2_ adsorption performance [55, 96, 181]. For instance, they manufactured two types of these structures using UTSA-16(Co) and MOF-74(Ni) as porous adsorbents and evaluated their adsorption capability for CO_2_ capture from air. The UTSA-16(Co) and MOF-74(Ni) monoliths with high particle loading of up to 85% and 80%, respectively, were prepared via a simple two-solution-based method (Fig. 23) [55]. The results demonstrated that the fabricated MOF monoliths retain their mechanical integrity and physical properties. However, their surface areas reduced significantly, as about 30% (from 631 to 444 m^2^ g^−1^) for UTSA-16(Co) and 38% (from 1180 to 737 m^2^ g^−1^) for MOF-74(Ni) monoliths, which may be related to the inclusion of different compounds (e.g., PVA and bentonite clay) employed to form these monoliths. This phenomenon may also be due to the component's contact with the aqueous solution during the paste preparation. The CO_2_ adsorption results showed that the 3D-printed UTSA-16(Co) and MOF-74(Ni) monoliths can adsorb CO_2_ molecules at room temperature and 500 ppm (0.5%) with adsorption capacities of 1.31 and 1.35 mmol g^−1^, respectively (Fig. 23b), which are 87% and 79% of the adsorption capacities of their pure MOFs at the similar situations.

In a subsequent study, they fabricated 3D-printed amine- (polyethylenimine (PEI) and tetraethylenepentamine (TEPA)) impregnated MOF monoliths and systematically investigated their CO_2_ adsorption performances for the elimination of this gas from a confined space [181]. The MOF monoliths were functionalized via pre- and post-synthetic approaches, in which for pre-functionalization the MIL-101 particles were immersed with TEPA or PEI and printed to create the monoliths. In post-functionalization strategy, the MOF powder was directly printed and then impregnated the monoliths with PEI or TEPA. The experimental CO_2_ adsorption results revealed that all amino MOFs-impregnated monoliths had increased CO_2_ adsorption capabilities compared to untreated monoliths. In particular, the pre-functionalization strategy yielded higher CO_2_ adsorption capacities than post-functionalization. However, despite high CO_2_ adsorption capacities of 3D-printed amino functional MIL-101 monoliths, they showed relatively slow adsorption kinetics, particularly for the pre-functionalization monoliths, since the rate of adsorption was restricted with the penetration of CO_2_ molecules into the monolith’s surfaces.

In the last decade, the MOF containing MMMs have received much interest in gas separation, mainly due to their facile processability and tremendous separation performance [182]. This improvement in the gas separation performance of MMMs can be further enhanced by the fabrication of thin-film composite (TFC) membranes because the thin membranes showed higher gas permeation without reducing the gas selectivity [183]. However, fabricating defect-free and continuous MOF-based TFC MMMs is a significant issue. Compared to the traditional TFC MMMs fabrication techniques (e.g., interfacial polymerization, spin coating, knife coating, dip coating, bar coating, and slot-die coating), the design, thickness, structure, and roughness of the manufactured membranes might be more precisely controlled using the 3D printing technology. Additionally, 3D membrane printing could present more highly favorable advantages for fabrication and commercialization, including quality, precision, speed, consistency, and cost [184].

**Fig. 24:**
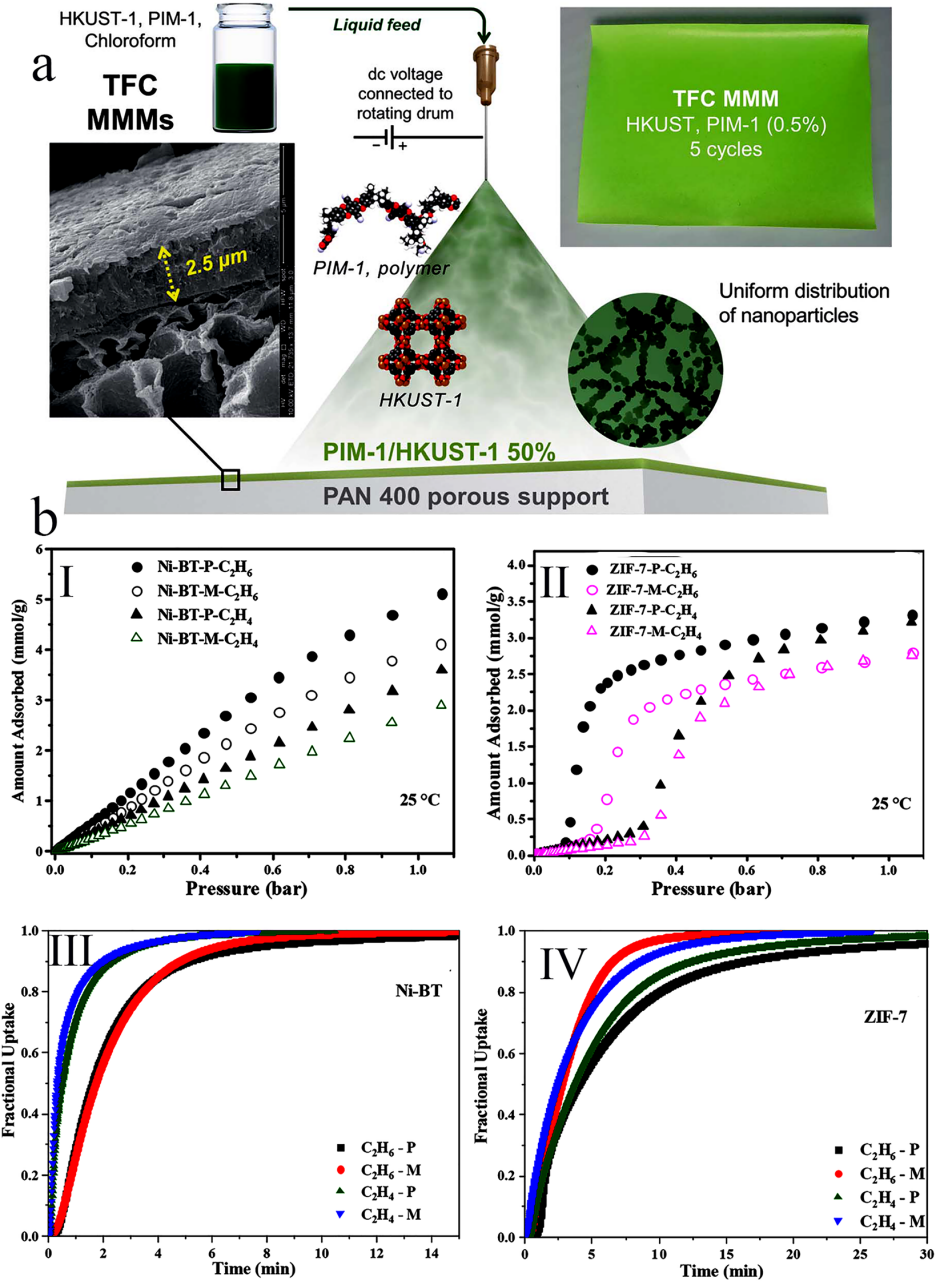
**a** Schematic illustration of the electrospray 3D printing method. Reprinted with permission from Ref. [185]. Copyright 2021, Royal Society of Chemistry. **b** Ethane/ethylene adsorption isotherms of (I) Ni(bdc)(ted)_0.5_ and (II) ZIF-8 monoliths and their particles at room temperature from 0 to 1 bar. Fractional uptake curves for ethane and ethylene over (III) Ni(bdc)(ted)_0.5_ and (IV) ZIF-8 monoliths at ambient conditions. Reprinted with permission from Ref. [97]. Copyright 2018, American Chemical Society

Based on this strategy, Elsaidi et al. [185] fabricated a series of 3D-printed TFC MMMs that included HKUST-1 as a highly porous filler and a microporous polymer (PIM-1) as a continuous matrix employing the electrospray printing method (Fig. 24a). They systematically investigated the influence of electrospray cycle number and casting concentration on the CO_2_ separation efficiency and membrane thickness. Accordingly, a low concentration of the PIM-1/HKUST-1 solution (0.1 wt%) was used to fabricate TFC membranes with a thickness smaller than 500 nm that exhibited poor CO_2_/N_2_ selectivity. However, by increasing the solution concentration (≈ 0.5 wt%), it was possible to fabricate TFC MMMs with a thickness of 2–3 µm that demonstrated not only greater CO_2_ permeation than pure PIM-1 membranes but also exhibited notable increases in CO_2_/N_2_ selectivity compared to the thinner membranes.

### H_2_ Adsorption/Separation

Hydrogen (H_2_) has been regarded as a green and renewable fuel source and is often used as a precursor in various industrial petrochemical processes [186]. Additionally, due to its big energy density and the possibility of producing it in a renewable manner, it is very appealing as a clean transportation fuel [187]. H_2_ can be produced via different methods, one of which is using solar energy to turn liquid water into H_2_ gas. However, the main drawback of developing this technology is the need for specialized H_2_ storage devices [188]. Therefore, developing new adsorbent materials for H_2_ storage with high adsorption uptake is critical.

Adsorbent materials to store H_2_ for mobile applications have been widely investigated over the past years. It has been reported that some MOFs show excellent H_2_ storage capacities [189]. MOF-5 with formula unit of Zn_4_O(BDC)_3_ (BDC = benzodicarboxylate), is among the earliest MOFs whose adsorption performance toward H_2_ storage has been well characterized [190]. However, the main drawback of this MOF for H_2_ storage applications is its complicated processability. Therefore, extensive efforts have been made to fabricate new MOF-based composite materials with improved H_2_ storage capacity and processability [189, 191]. Kreider et al. [59] incorporated MOF-5 into an ABS composite through a conventional thermoplastic 3D printer to address this challenge. 3D-printed ABS-MOF-5 composites could be printed into various geometries at MOF-5 loadings of 10% or less (Fig. 10b). It was observed that some of the MOF-5 nanoparticles were degraded in the mixing procedure, mostly owing to moisture during the purifying and solvent-casting steps. However, despite this partial degradation, the MOF-5 particles maintain their ability to H_2_ storage, in which the H_2_ storage capacities of ABS-MOF-5 composites are relatively higher than that of the pure ABS matrix.

The other main way for H_2_ production is steam reforming of different hydrocarbons [192]. Generally, this process creates a mixture of H_2_, CO_2_, and CO, which contains inert gases that can decrease its market value and energy density. CO_2_, as an inert gas, is utilized in catalytic reactions to a smaller extent and is typically more difficult to employ, thereby, its content in exhaust steams is normally larger than that of CO. Thus, separation of CO_2_ from H_2_ is a critical step in H_2_ upgrading. Among numerous reported MOFs, MOF-74(Ni) is one of the promising candidates for H_2_ upgrading via CO_2_/H_2_ separation due to its negligible affinity toward H_2_ molecules and large micropore volume, which allow it to store a large amount of CO_2_ molecules without compromising the H_2_ recovery [193]. However, before utilizing this MOF in pressure swing adsorption (PSA) processes, particle shape should be engineered into contactors to decrease the amount of scattering, enhance heat/mass transfer, and improve column packing. In this regard, Lawson and Rezaei [194] structured this MOF into honeycomb monoliths via 3D printing. They investigated the CO_2_/H_2_ separation performance of the fabricated monoliths at different superficial velocity, adsorption time, adsorption pressures, and feed compositions. The breakthrough experiments revealed that the higher pressure improves the amount of CO_2_/H_2_ wavefront separation while raising the superficial velocity results in a wider range of breakthrough profiles. Additionally, it showed that enhancing the CO concentration results in greater competitive adsorption with CO_2_ molecules and wider wavefronts. This research revealed a basic perspective into the adsorption efficiency of 3D-printed MOF-74(Ni) monoliths for CO_2_/N_2_ separation, which could benefit future scale-up gas separation applications.

### Adsorption/Separation of Other Gases

Light olefins like ethylene and propylene are critical precursors for numerous vital chemicals and products. However, for the synthesis of special chemicals or polymers from olefins, olefin must be produced with extremely high purity (> 99.9%). Therefore, in the chemical industry, efficient techniques to separate olefins from paraffins are very important [195]. Since ethylene and ethane have comparable volatilities and dimensions, they are normally separated from the off-gas stream using a very energy-consuming cryogenic distillation process [196]. In recent years, adsorption separation of light olefins from related paraffin using MOF-based adsorbent materials has received a lot of interest [197].

As a representative study, Dhainaut et al. [105] used a 3D printer approach to manufacture a series of MOF-based adsorbent materials with regulated morphologies using shear-thinning inks including 2-hydroxyethyl cellulose as binder and PVA as plasticizer. Four different MOF-based adsorbent materials with controlled macroscale morphology were prepared using four benchmark MOFs, including CPL-1, UiO-66-NH_2_, ZIF-8, and HKUST-1. All the fabricated 3D-printed MOF-based adsorbent materials were physically stable during uniaxial compression of up to 0.6 MPa and extremely porous, with specific surface areas decreased by 0 to -25%. Moreover, these adsorbent materials were used for high-pressure adsorption of certain hydrocarbons (e.g., C_2_H_6_, CH_4_, and C_2_H_4_), and some of them (e.g., ZIF-8, HKUST-1, and UiO-66-NH_2_) showed great methane storage ability, and the other (CPL-1) exhibited an excellent ethane/ethylene separation performance.

In another effort, Thakkar et al. [97] employed DIW strategy to produce MOF monoliths that include 80 wt% Ni(bdc)(ted)_0.5_ (ted = triethylenediamine) and 85 wt% ZIF-7, then have subsequently examined their adsorption performance toward ethane/ethylene separation. Because of the gate-opening action of ZIF-7 framework, it enables to selective and dynamic separation ethylene and ethane at relatively low pressure (Fig. 24b(I)). While the Ni(bdc)(ted)_0.5_ monolith exhibited higher selectivity toward ethane than ethylene at higher pressures. This observation may be due to the pore window of this MOF, which is sufficiently broad to adsorb ethane molecules but does not impart sufficient electrostatic interactions to adsorb the relatively smaller ethylene molecules (Fig. 24b(II)). Accordingly, the maximum adsorption capacities of ZIF-7 monolith for C_2_H_4_ and C_2_H_6_ gases were found to be about 2.75 and 2.8 mmol g^−1^, respectively, at room temperature and 1 bar. The Ni(bdc)(ted)_0.5_ monolith exhibited relatively greater uptakes of 2.9 and 4.2 mmol g^−1^ toward C_2_H_4_ and C_2_H_6_ gases, respectively.

As observed from Fig. 24b (III and IV), both fabricated 3D-printed monoliths exhibited higher breakthrough times for ethane than ethylene in the dynamic tests, demonstrating that both adsorbents were capable of separating the paraffin/olefin mixture with good selectivity. These distinctions in breakthrough time for ethylene and ethane were larger for Ni(bdc)(ted)_0.5_ monolith. Moreover, the ideal adsorption solution theory (IAST) approach was used to estimate C_2_H_6_/C_2_H_4_ selectivity for Ni(bdc)(ted)_0.5_ and ZIF-7 monoliths, which were in the range of 1.2–2.0 and 1.9–11.8, respectively. Furthermore, it was found that these two monoliths exhibited higher breakthrough fronts compared to their pure MOF, indicating that structuring MOF particles by DIW method can be applied to improve their mass transfer capabilities.

## Conclusion and Perspective

The shaping of MOF powders into MOF-based macroscopic materials is an essential way to promote their industrial practicability. Also, it is needed to overcome the challenges related to their powders (e.g., abrasion, dust formation, clogging, toxicity, pressure drop, agglomeration, and difficult separation and recycling). Nowadays, many shaping technologies, including palletization, granulation, and converting powders into thin films, are existed for shaping MOFs. For a certain application, each shaping procedure delivers distinct features to the finished items in terms of size, morphology, and appearance. However, these shaping methods still have disadvantages, including significant surface area reduction and pressure drop issues when applied as adsorbent for adsorption/separation applications.

3D printing technology has been extensively applied to convert MOF powders into robust 3D-printed MOF monoliths with tunable morphology for favorable applications. This review summarizes the recent progress associated with 3D-printed MOF monoliths, including the selection and optimization of the fabrication conditions and excellent performance improvement compared to those of the powdered forms. Hence, we have summarized the main important fabrication strategies such as direct ink writing (DIW), seed-assisted in situ growth, coordination replication from solid precursors, matrix incorporation, selective laser sintering (SLS), and digital light processing (DLP) for the construction of 3D-printed MOF monoliths. Accordingly, for a specific MOF, it is very important to select and optimize the suitable fabrication strategy based on its inherent properties and practical application requirements to manufacture appropriate 3D-printed MOF monoliths for applications in various industrial fields, including detection of water pollutants, removal of heavy metal ions and organic dyes, separation of oily compounds from contaminated water, serving as catalyst/photocatalyst for degradation of organic dyes, CO_2_ capture, and adsorption/separation of other gases.

These requirements stimulate the development of 3D-printed MOF monoliths because of structure, morphology, composition, and functions concerning the following aspects: low fabrication cost, moderate to low toxicity, easy handling of the final objects, high specific surface area as well as high porosity, sufficient compression resistance, high adsorption capacity as applied as adsorbent, good performance in various fields, high chemical/thermal/mechanical stability, high MOF loading capacity, facile regeneration, and excellent recyclability. Therefore, by focusing on various fabrication strategies, we have presented deep insights into the structures and textural features of 3D-printed MOF monoliths. Moreover, the relationship between the microscopic structure of 3D-printed MOF monoliths and their macroscopic performances has also been explored.

Although many advances have been achieved in the shaping of MOF powders via 3D printing technology, the 3D-printed MOF monoliths are still in their infancy, and some technical obstacles must be overcome in future works to realize their practical applicability. (i) MOF toxicity: although the metal ions or organic ligand leaching from MOFs has been significantly reduced after converting them into 3D-printed MOF monoliths, secondary water pollution because of the poor stability and durability of MOFs is still a main issue in water treatment processes. Thus, MOFs with good structural stability are needed to overcome this challenge. (ii) MOF structural stability remains unsatisfactory, particularly mechanical, thermal, and chemical stability. (iii) At present, several well-known MOFs, including ZIF-8, HKUST-1, UiO-66 family, and the MIL-n family, are extensively reported 3D-printed MOF monoliths. However, many kinds of MOFs have developed in recent years, which will make progress in the field of 3D-printed MOF monoliths. (iv) The cost of precursors and fabrication method of 3D-printed MOF monoliths: the large-scale application of 3D-printed MOF monoliths requires the design of low-cost, simple, sustainable, rapid, and highly efficient fabrication methods. New synthesis techniques using low-priced raw materials and green solvents like water will reduce the overall cost. A novel fabrication strategy using stable MOFs capable of regeneration and recycling can also reduce costs. The rational design and synthesis of polymers and MOF particles with low-cost materials and without using a solvent, which resulted in the fabrication of robust 3D-printed MOF monoliths, is a promising area where attention can be focused in the future. (v) At present, most of the MOF shaping strategies are still in the laboratory stage, while the large-scale production of 3D-printed MOF monoliths is a critical step in their commercialization and practical applications. (vi) Until recently, various manufacturing processes for 3D-printed MOF monoliths have been devised and tested; however, the synthesis mechanism remains unclear. Therefore, further studies on the exploration of synthesis mechanisms and the relationship between the microstructure and activity of 3D-printed MOFs are needed for mass production. Consequently, the fabrication of 3D-printed MOF monoliths and their potential applications still involves many challenges and opportunities. However, we strongly believe that the 3D-printed MOF monoliths should present an encouraging future with persistent efforts toward these challenges.

## Abbreviations

^131^I: Radioiodine
3D: Three-dimensional
ABS: Acrylonitrile butadiene styrene
ALD: Atomic layer deposition
AO: Auramine O
ASV: Anodic striping voltammetry
BDC: 1,4-Benzenedicarboxylate
BET: Brunauer–Emmett–Teller
BTC: 1,3,5-Benzenetricarboxylic acid
CA-GE: Calcium alginate and gelatin
CE: Counter electrode
COFs: Covalent organic frameworks
DIW: Direct ink writing
DLP: Digital light processing
DN: Double network
DR31: Direct red 31
EDS: Energy-dispersive X-ray spectroscopy
FDM: Fused deposition modeling
FESEM: Field emission scanning electron microscopy
GCE: Glassy carbon electrode
GP: Graphite paste
GPG: Gel-print-grow
HKUST: Hong Kong University of Science and Technology
Hmim: 2-Methylimidazole
HPC: Hierarchical porous ceramics
IAST: Ideal adsorption solution theory
Ln-MOFs: Lanthanide-organic frameworks
MB: Methylene blue
MG: Malachite green
MMFs: Mixed matrix films
MMMs: Mixed-matrix membranes
MOFs: Metal-organic frameworks
MV: Methyl violet
NPs: Nanoparticles
PA: Polyamide
PCPs: Porous coordination polymers
PDMS: Polydimethylsiloxane
PEI: Polyethylenimine
PEO: Polyethylene oxide
PIM-1: Polymer of intrinsic microporosity
PLA: Poly(lactic acid)
PSA: Pressure swing adsorption
PVA: Poly(vinyl alcohol)
PVDF: Polyvinylidene fluoride
PVDF-HFP: Poly(vinylidene fluoride-co-hexafluoropropylene)
PVP: Poly(vinylpyrrolidone)
R6G: Rhodamine 6G
RB: Rodamine B
RE: Reference electrode
ROS: Reactive oxygen species
SA-GE: Sodium alginate and gelatin
SLS: Selective laser sintering
STP: Standard temperature and pressure
TEA: Triethylamine
ted: Triethylenediamine
TEM: Transmission electron microscopy
TEPA: Tetraethylenepentamine
TFC: Thin-film composite
TMPPTA: Trimethylolpropane propoxylate triacrylate
TOCNF: 2,2,6,6-Tetramethylpiperidine-1-oxylradical-mediated oxidized cellulose nanofibers
TPU: Thermoplastic polyurethane
UiO: University of Oslo
UV: Ultraviolet
WE: Working electrode
WHO: World Health Organization
XRD: X-ray diffraction
XRPD: X-ray powder diffraction patterns
ZIF: Zeolite imidazole framework
